# Effectiveness assessment of using riverine water eDNA to simultaneously monitor the riverine and riparian biodiversity information

**DOI:** 10.1038/s41598-021-03733-7

**Published:** 2021-12-20

**Authors:** Haile Yang, Hao Du, Hongfang Qi, Luxian Yu, Xindong Hou, Hui Zhang, Junyi Li, Jinming Wu, Chengyou Wang, Qiong Zhou, Qiwei Wei

**Affiliations:** 1 Key Laboratory of Freshwater Biodiversity Conservation, Ministry of Agriculture and Rural Affairs of China, Yangtze River Fisheries Research Institute, Chinese Academy of Fishery Sciences, Wuhan, 430223 China; 2 Qinghai Key Laboratory of Qinghai-Lake Naked Carps Breeding and Conservation, Rescue and Rehabilitation Center of Naked Carps of Qinghai Lake, Xining, 810016 China; 3 State Key Laboratory of Biogeology and Environmental Geology, China University of Geoscience, Wuhan, 430074 China

**Keywords:** Ecology, Ecology, Environmental sciences

## Abstract

Both aquatic and terrestrial biodiversity information can be detected in riverine water environmental DNA (eDNA). However, the effectiveness of using riverine water eDNA to simultaneously monitor the riverine and terrestrial biodiversity information remains unidentified. Here, we proposed that the monitoring effectiveness could be approximated by the transportation effectiveness of land-to-river and upstream-to-downstream biodiversity information flows and described by three new indicators. Subsequently, we conducted a case study in a watershed on the Qinghai–Tibet Plateau. The results demonstrated that there was higher monitoring effectiveness on summer or autumn rainy days than in other seasons and weather conditions. The monitoring of the bacterial biodiversity information was more efficient than the monitoring of the eukaryotic biodiversity information. On summer rainy days, 43–76% of species information in riparian sites could be detected in adjacent riverine water eDNA samples, 92–99% of species information in riverine sites could be detected in a 1-km downstream eDNA sample, and half of dead bioinformation (the bioinformation labeling the biological material that lacked life activity and fertility) could be monitored 4–6 km downstream for eukaryotes and 13–19 km downstream for bacteria. The current study provided reference method and data for future monitoring projects design and for future monitoring results evaluation.

## Introduction

Biodiversity monitoring is the basis of ecological research, biodiversity conservation and ecosystem management^1,2^. Traditional biodiversity monitoring methods are cost- and time-consuming and require high levels of expertise, in which biodiversity is often studied from a local and low spatio-temporal resolution perspective and is generally not available at a wide taxonomic breadth, high spatio-temporal resolution and large spatio-temporal scale^3–5^. This limits the development of ecological research, biodiversity conservation and ecosystem management. Currently, metabarcoding and high-throughput sequencing of environmental DNA (eDNA, DNA extracted from environmental samples such as water, soil, and air) provide novel opportunities to monitor biodiversity^5–9^. As an efficient and easy-to-standardize non-invasive monitoring approach^6,10–12^, and with the continuous advancements in DNA sequencing technology, using eDNA metabarcoding to monitor biodiversity is an appropriate method to revolutionize biodiversity monitoring by enabling the census of wide taxonomic species on a high spatio-temporal resolution and large spatio-temporal scale^4,6,13,14^. Streams and rivers connect upstream and downstream regions, connect land with waterbodies, and transport materials and information through extensive and heterogeneous network systems^6,15,16^. Riverine water eDNA incorporates biodiversity information across terrestrial and aquatic biomes^6,16^. Therefore, samples of riverine water eDNA have the potential to simultaneously monitor both aquatic and terrestrial biodiversity information of a watershed for biodiversity research, conservation, and management. However, its viability and monitoring effectiveness (represented by the proportion of aquatic and terrestrial biodiversity information that can be detected by using limited riverine water eDNA samples) has not been systematically identified.

The effectiveness of using riverine water eDNA to simultaneously monitor both aquatic and terrestrial biodiversity depends on the land-to-river and upstream-to-downstream transportation effectiveness of the terrestrial and upstream biodiversity information^6,17–20^. The biodiversity information monitoring effectiveness could be approximated by assessing the land-to-river and upstream-to-downstream transportation effectiveness of the corresponding bioinformation (eDNA). Here we defined the land-to-river and upstream-to-downstream bioinformation transportation (including organisms, nucleic acids, peptides and other biomarkers), which is driven by the hydrologic processes of watershed systems, as the watershed biological information flow (WBIF). WBIF integrates the ecological processes of eDNA, including the origin, state, transport, and fate of eDNA^14,15,21–23^. The transportation effectiveness of WBIF mainly relies on the transport capacity, degradation rate, and environmental filtration of WBIF^15,21–23^. The transport capacity of WBIF mainly depends on erosion and runoff^12,15,24^. Additionally, the degradation rate of WBIF mainly depends on environmental features^21,25,26^, and the environmental filtration of WBIF mainly depends on the environmental changes of restricting organisms. Collectively, all of these factors are related to the seasons and weather conditions^26^. Therefore, we hypothesized that the monitoring effectiveness of riverine water eDNA would vary with the seasons and weather conditions. Moreover, due to taxonomy-specific eDNA degradation rates^27^, species-specific eDNA degradation rates^17^, and form-specific eDNA degradation rates^28^, we hypothesized that the monitoring effectiveness of riverine water eDNA would vary with taxonomic communities.

Herein, we proposed that, in order to identify the effectiveness of using riverine water eDNA to simultaneously monitor the riverine and terrestrial biodiversity information, we needed to assess the transportation effectiveness of land-to-river and upstream-to-downstream WBIF for different taxonomic communities in different seasons and weather conditions. In the present study, we conducted a case study in a watershed on the Qinghai–Tibet Plateau to test the eDNA monitoring effectiveness assessment framework. We estimated the monitoring effectiveness, as indicated by the biodiversity information of three taxonomic communities in three seasons and weather conditions. Our objectives were threefold: (1) to identify the variation in biodiversity information monitoring effectiveness in different seasons and weather conditions; (2) to identify the variation in the effectiveness for monitoring the biodiversity information of different taxonomic communities; and (3) to test the monitoring effectiveness assessment framework.

## Results

### WBIF of the three seasonal groups

**Figure 1:**
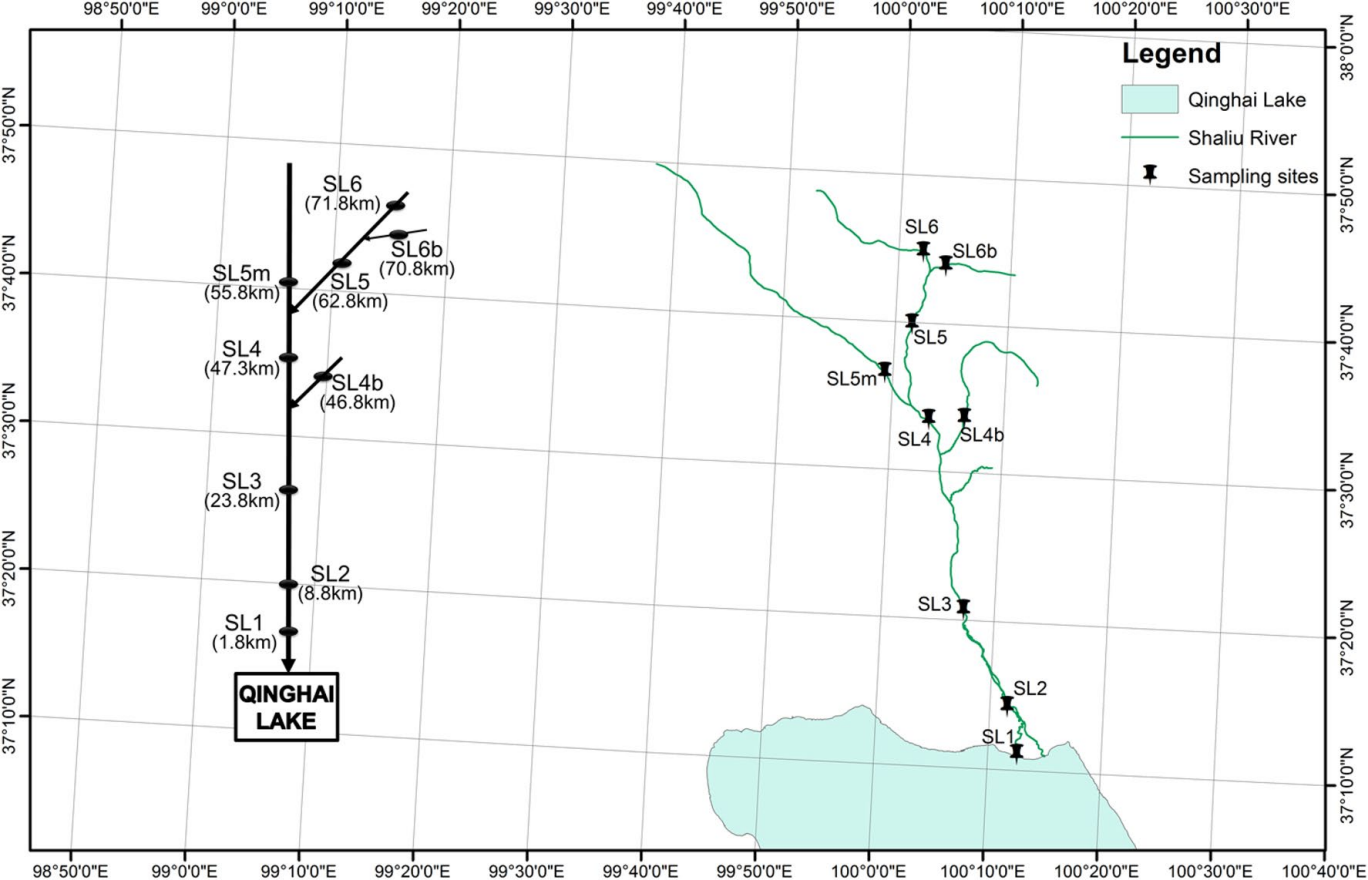
Sampling transects. SL1 denotes the first sampling transect on the Shaliu River. The distances labeled in parentheses under the tags of sampling transects denote the distances from the estuary to the sampling transects, such as SL1 (1.8 km), which means the distance from the estuary to SL1 is 1.8 km.

**Figure 2:**
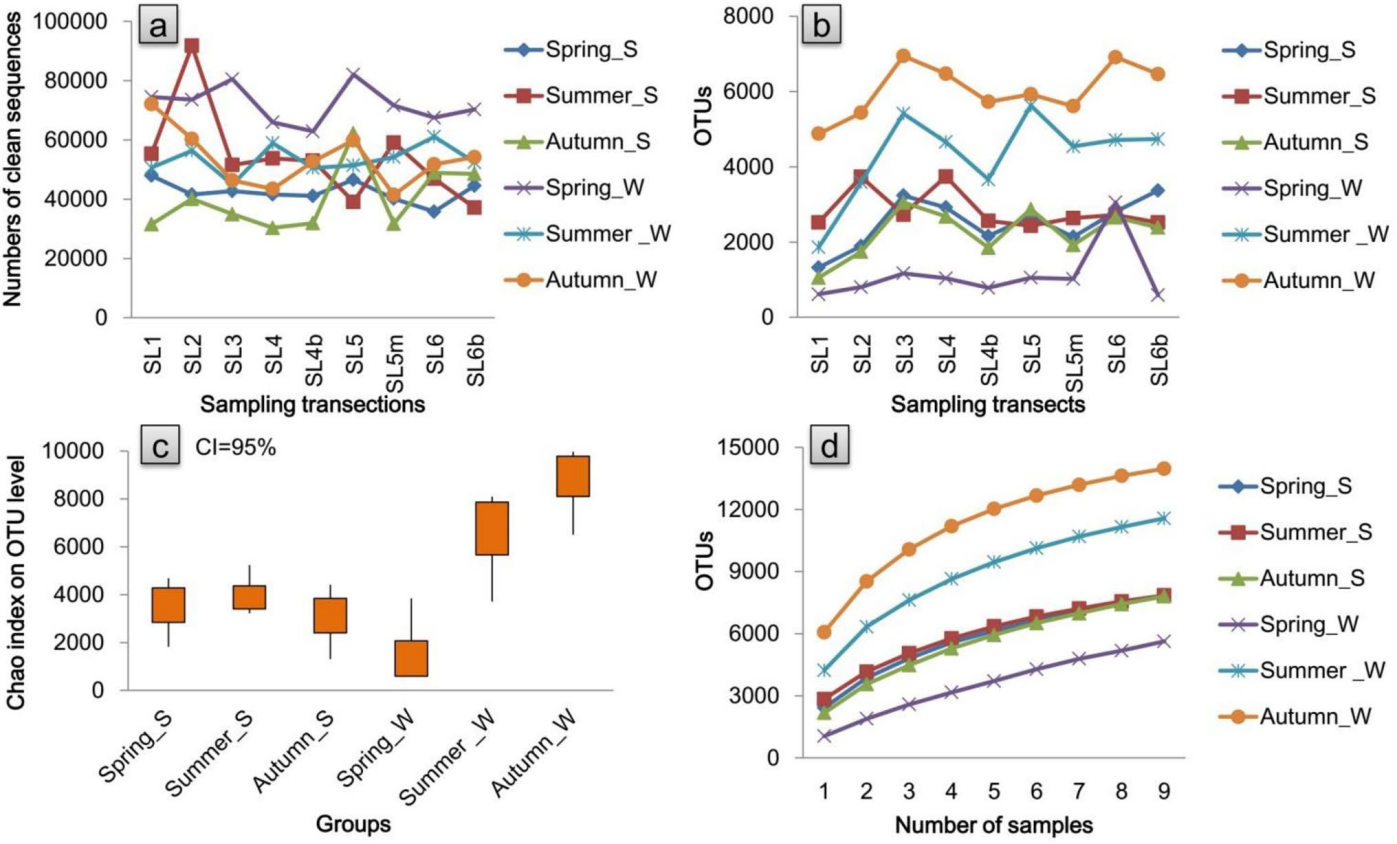
Biological information features of the samples: numbers of clean sequences in each sample (**a**), OTUs in each sample (**b**), community richness of each sample at the OTU level (**c**) and species accumulation curves at the OTU level (**d**). Spring_S denotes the riparian soil eDNA samples that were sampled during April 2019; Spring_W denotes the riverine water eDNA samples that were sampled during April 2019; Summer_S denotes the riparian soil eDNA samples that were sampled during June 2019; Summer_W denotes the riverine water eDNA samples that were sampled during June 2019; Autumn_S denotes the riparian soil eDNA samples that were sampled during September 2019; Autumn_W denotes the riverine water eDNA samples that were sampled during September 2019.

**Figure 3:**
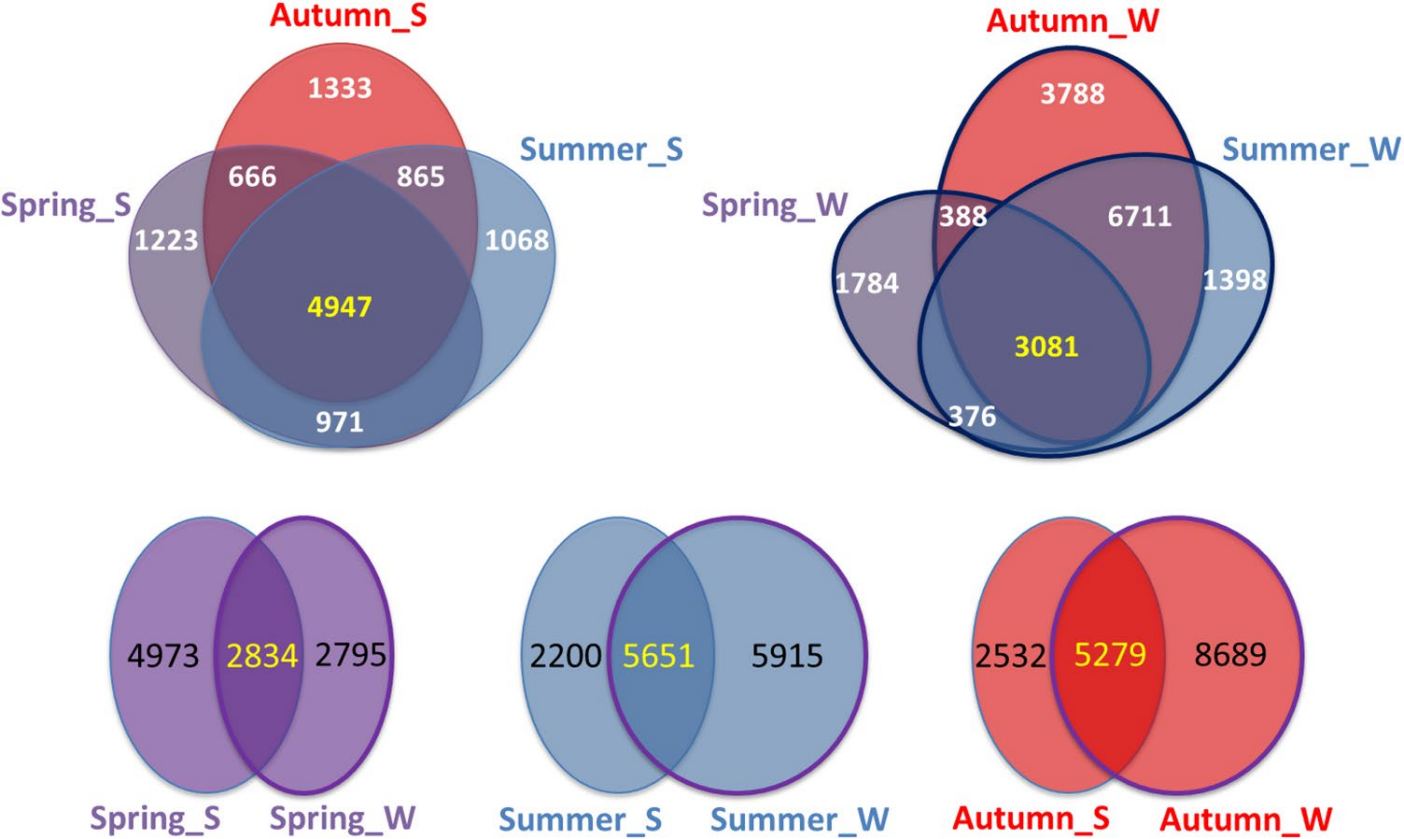
OTUs in riparian soil samples (S) and riverine water samples (W) shared by the three groups (spring, summer and autumn). Spring_S denotes the riparian soil eDNA samples that were sampled during April 2019; Spring_W denotes the riverine water eDNA samples that were sampled during April 2019; Summer_S denotes the riparian soil eDNA samples that were sampled during June 2019; Summer_W denotes the riverine water eDNA samples that were sampled during June 2019; Autumn_S denotes the riparian soil eDNA samples that were sampled during September 2019; Autumn_W denotes the riverine water eDNA samples that were sampled during September 2019. The circle that indicates the riverine water samples has a line, the circle that indicates the riparian soil samples do not have a line. The numbers in the circles denote the OTUs.

A total of 10,602, 13,766, and 16,500 bacterial OTUs were detected from the samples (including 9 riverine water samples and 9 riparian soil samples, Fig. 1) of the spring group (sampling on frozen days), summer group (sampling on rainy days) and autumn group (sampling on cloudy days), respectively (Fig. 2, Supplementary Fig. S1 and Supplementary Tables S1, S2). The total OTUs that were detected from the riparian soil eDNA samples were similar among the seasons (Figs. 2, 3, Supplementary Fig. S1). The total OTUs that were detected from the riverine water eDNA samples were richest in the autumn (Fig. 2, 3, Supplementary Fig. S1). The common OTUs that were shared between the riparian soil eDNA and riverine water eDNA samples accounted for 36.30%, 71.98%, and 67.58% of the total OTUs that were detected in the riparian soil eDNA samples in the spring, summer, and autumn groups, respectively (Fig. 3).

The transportation effectiveness values of WBIF, as indicated by bacterial OTUs from the riparian sampling site to the adjacent riverine sampling site, were 16.62%, 62.76%, and 48.09% on spring frozen, summer rainy, and autumn cloudy days, respectively, among which there was the highest transport capacity and the lowest environmental filtration on the summer rainy day (Table 1, Supplementary Table S3). The transportation effectiveness of WBIF indicated by bacterial OTUs from upstream to downstream was 75.86%, 97.41%, and 96.07% per km on spring frozen, summer rainy, and autumn cloudy days, respectively (Table 2, Supplementary Table S4), among which the transport capacity was more than 99% in all three seasons and the least noneffective WBIF (dead bioinformation) occurred; the longest half-life distance of the noneffective WBIF occurred on the summer rainy day (Table 2).

**Table 1:** Seasonal variation of transport capacity, environmental filtration, and transportation effectiveness of watershed biological information flow (WBIF) from the riparian sampling site to adjacent riverine water sampling site in three seasons indicated by bacterial OTUs.

Seasonal group	Weather condition	Transport capacity	Environmental filtration	Transportation effectiveness
Spring group	Frozen days	0.268791 ± 0.202388	0.385443 ± 0.029320	0.166152 ± 0.125394
Summer group	Rainy days	0.684876 ± 0.091302	0.083816 ± 0.020574	0.627643 ± 0.087327
Autumn group	Cloudy days	0.573579 ± 0.052897	0.161800 ± 0.045075	0.480933 ± 0.052179

The spring group was sampled during April 2019; the summer group was sampled during June 2019; the autumn group was sampled during September 2019. Statistics for the spring group are based on 8 sampling transects except estuary (SL1); statistics for the summer and autumn groups are based on 7 sampling transects except two downstream transects (SL1 and SL2). CI = 95%.

**Table 2:** Seasonal variation of transport capacity, proportion of noneffective WBIF, half-life distance of the noneffective WBIF, and transportation effectiveness of watershed biological information flow (WBIF) from the upstream to downstream regions indicated by bacterial OTUs.

Seasonal group	Weather condition	Transport capacity (per km)	Proportion of noneffective WBIF	Half-life distance of the noneffective WBIF	Transportation effectiveness (per km)	Environmental filtration from rain point to sunny point	Environmental filtration from freshwater to saline-water
Spring group	Frozen days	0.999706 ± 0.000305	0.668465 ± 0.003435	1.548987 ± 0.126870	0.758618 ± 0.000304	/	0.160427 ± 0.008244
Summer group	Rainy days	0.994245 ± 0.000941	0.434635 ± 0.041681	14.52338 ± 1.440539	0.974105 ± 0.000926	0.005687 ± 0.005450	0.544164 ± 0.010042
Autumn group	Cloudy days	0.992250 ± 0.001452	0.493504 ± 0.041043	10.398112 ± 0.711122	0.960671 ± 0.001415	/	0.128718 ± 0.017062

The spring group was sampled during April 2019; the summer group was sampled during June 2019; the autumn group was sampled during September 2019. CI = 95%.

### WBIF of the three taxonomic groups

**Figure 4:**
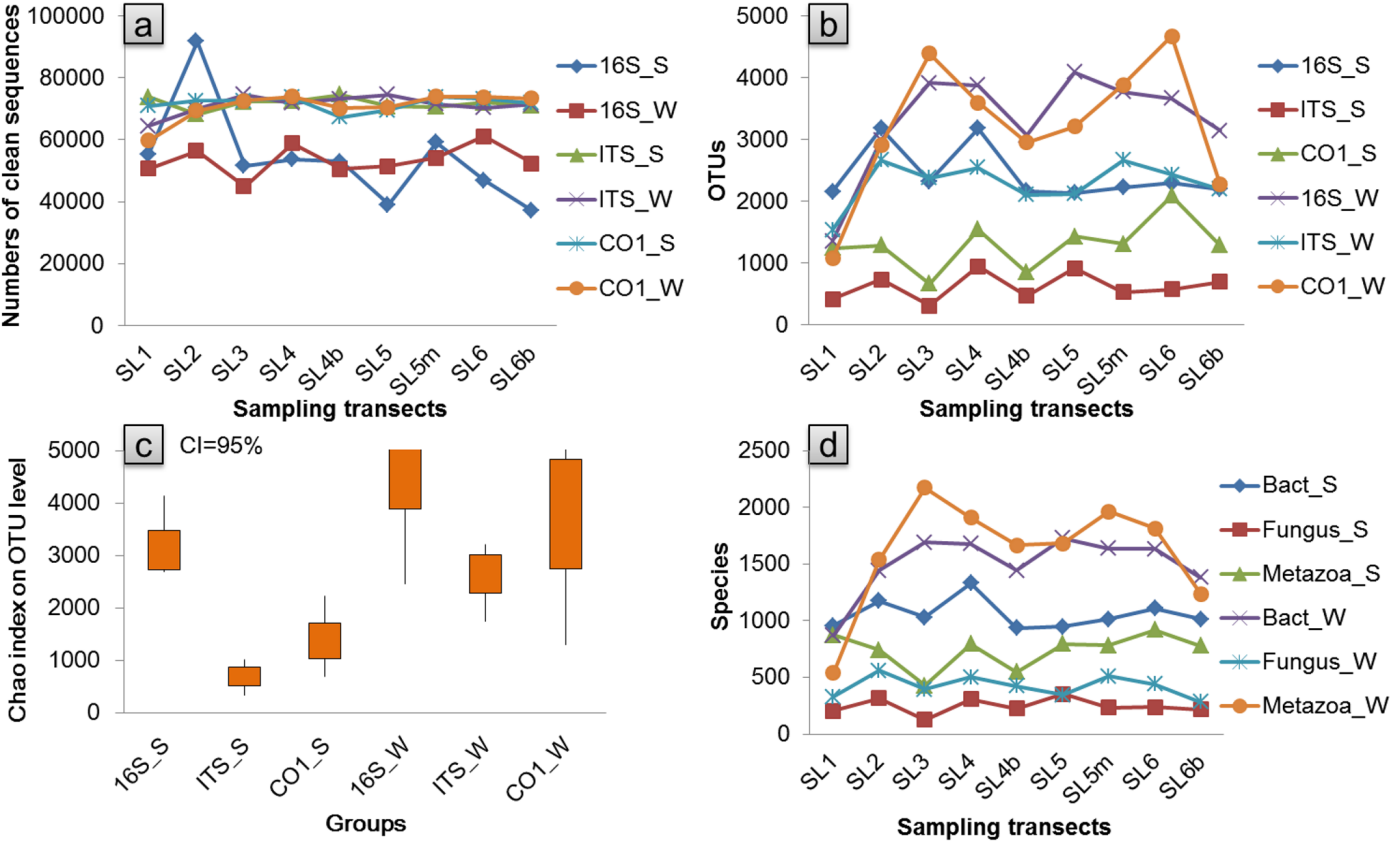
Biological information features of the samples: numbers of clean sequences in each sample (**a**), OTUs in each sample (**b**), community richness of each sample at the OTU level (**c**), and species in each sample (**d**). 16S_S denotes the riparian soil eDNA samples that were sequenced using the bacterial 16S rRNA gene; ITS_S denotes the riparian soil eDNA samples that were sequenced using the fungal ITS gene; CO1_W denotes the riverine water eDNA samples that were sequenced using the eukaryotic mitochondrial CO1 gene. Bac_S denotes the bacterial group detected in the riparian soil eDNA samples; Fungus_S denotes the fungal group detected in the riparian soil eDNA samples; and Metazoa_W denotes the metazoan group detected in the riverine water eDNA samples.

A total of 13,766, 7098, and 17,316 kinds of OTUs and 3532, 1032, and 6836 kinds of species were detected among the 18 summer samples, as indicated by the 16S rRNA gene, ITS gene, and CO1 gene, respectively (Fig. 4, Supplementary Fig. S2 and Supplementary Table S5). The OTUs and species detected in the riverine water eDNA samples were generally higher than in the riparian soil eDNA samples for all three taxonomic communities (Fig. 4). The common OTUs and species shared between the riparian soil and riverine water eDNA samples accounted for 71.98% and 87.95%, 60.40% and 76.18%, and 37.93% and 53.52% of the total OTUs and species in the bacterial, fungal and eukaryotic group, respectively.

The transportation effectiveness of the bacterial, fungal, and eukaryotic WBIF from the riparian sampling site to the adjacent riverine sampling site was 62.76%, 44.79%, and 22.64% at the OTU level, respectively, and 80.75%, 65.62%, and 43.38% at the species level, respectively, among which both the transport capacity and environmental filtration significantly declined with the bacterial, fungal, and eukaryotic communities (Table 3, Supplementary Tables S6, S7). The transportation effectiveness of bacterial, fungal and eukaryotic WBIF from upstream to downstream was 97.41%, 92.64%, and 89.83% per km at the OTU level, and 98.69%, 95.71%, and 92.41% per km at the species level, respectively, among which the noneffective WBIF decreased with the bacterial, fungal, and eukaryotic communities (Table 4, Supplementary Tables S8, S9), and the half-life distance of the noneffective WBIF was 14.52, 4.93, and 4.07 km at the OTU level and 17.82, 5.96, and 5.02 km at the species level for the bacterial, fungal, and eukaryotic groups, respectively (Table 4).

**Table 3:** Transport capacity, environmental filtration, and transportation effectiveness of watershed biological information flow (WBIF) from the riparian sampling site to the adjacent riverine water sampling site on summer rainy days, as indicated by three taxonomic groups.

Taxonomic group	Taxonomic level	Transport capacity	Environmental filtration	Transportation effectiveness
Bacteria (detected by the 16S rRNA gene)	OTU level	0.684876 ± 0.091302	0.083816 ± 0.020574	0.627643 ± 0.087327
	Species level	0.829912 ± 0.066079	0.027020 ± 0.007048	0.807461 ± 0.064521
Fungi (detected by the ITS gene)	OTU level	0.600756 ± 0.102865	0.258922 ± 0.054794	0.447896 ± 0.095670
	Species level	0.738975 ± 0.100006	0.113469 ± 0.016910	0.656191 ± 0.097099
Metazoan (detected by the CO1 gene)	OTU level	0.440871 ± 0.124206	0.485954 ± 0.061102	0.226403 ± 0.071669
	Species level	0.604263 ± 0.092950	0.281177 ± 0.028991	0.433842 ± 0.066684

Bacteria (detected by the 16S rRNA gene), fungi (detected by the ITS gene), and metazoans (detected by the CO1 gene) indicate the groups of bacteria (detected by the 16S rRNA gene), fungi (detected by the ITS gene), and metazoans (detected by the CO1 gene), respectively. Statistics in all groups are based on 7 sampling transects, except for two downstream transects (SL1 and SL2). CI = 95%.

**Table 4:** Transport capacity, proportion of noneffective WBIF, half-life distance of the noneffective WBIF, and transportation effectiveness of watershed biological information flow (WBIF) from the upstream to downstream regions on summer rainy days, indicated by three taxonomic groups at the OTU and species levels estimated by programming-solved according to the evolutionary algorithm.

Taxonomic group	Taxonomic level	Transport capacity (per km)	Proportion of noneffective WBIF	Half-life distance of the noneffective WBIF	Transportation effectiveness (per km)	Environmental filtration from rain point to sunny point	Environmental filtration from freshwater to saline-water
Bacteria (detected by the 16S rRNA gene)	OTU level	0.994245 ± 0.000941	0.434635 ± 0.041681	14.52338 ± 1.440539	0.974105 ± 0.000926	0.005687 ± 0.005450	0.544164 ± 0.010042
	Species level	0.998188 ± 0.000121	0.296484 ± 0.010590	17.82057 ± 1.215028	0.986898 ± 0.000121	0.051209 ± 0.005337	0.460245 ± 0.001469
Fungi (detected by the ITS gene)	OTU level	0.995550 ± 0.000680	0.529290 ± 0.016749	4.925445 ± 0.353730	0.926377 ± 0.000670	0.003482 ± 0.002886	0.338354 ± 0.003866
	Species level	0.999484 ± 0.000244	0.386710 ± 0.008333	5.961259 ± 0.264864	0.957057 ± 0.000242	0.000541 ± 0.000258	0.224685 ± 0.001239
Metazoan (detected by the CO1 gene)	OTU level	0.989275 ± 0.000923	0.587740 ± 0.019079	4.073058 ± 0.362046	0.898288 ± 0.000908	0.007897 ± 0.006958	0.716408 ± 0.003182
	Species level	0.992862 ± 0.000724	0.537202 ± 0.016816	5.018684 ± 0.317762	0.924058 ± 0.000713	0.005337 ± 0.002702	0.607287 ± 0.002642

Bacteria (detected by the 16S rRNA gene), fungi (detected by the ITS gene), and metazoans (detected by the CO1 gene) indicate the groups of bacteria (detected by the 16S rRNA gene), fungi (detected by the ITS gene), and metazoans (detected by the CO1 gene), respectively. CI = 95%.

## Discussion

Driven by the land-to-river and upstream-to-downstream WBIF, biodiversity information across terrestrial and aquatic biomes could be detected in riverine water eDNA^6,16^, and the monitoring effectiveness of riverine water eDNA relies on the transportation effectiveness of corresponding WBIF^6,17–20^. The transportation effectiveness of WBIF mainly relies on the transport capacity, degradation rate, and environmental filtration of WBIF^15,21–23^, which can vary with different seasons and weather conditions^26^. We hypothesized that the monitoring effectiveness would vary with the seasons and weather conditions. In the present case, the bacterial community richness in riparian soil did not vary with season, whereas the bacterial community composition in riverine water was richest in the autumn, followed by the summer (Figs. 2, 3). The transportation effectiveness of riparian-to-river and upstream-to-downstream WBIF in spring frozen days was significantly lower than in summer rainy days and autumn cloudy days (Tables 1, 2, Supplementary Tables S3, S4). Considering the insufficient read depth on the riverine water samples of summer and autumn groups (Supplementary Fig. S1), the riverine water bacterial community richness and the riparian-to-river transportation effectiveness on summer and autumn were already underestimated. It indicates that the monitoring effectiveness varied with different seasons and weather conditions, and summer and autumn were the optimal seasons, along with rainy days being the optimal weather condition, for using riverine water eDNA to simultaneously monitor the holistic biodiversity information in riverine sites and riparian sites.

The biodiversity information detected by water eDNA could originate from living and dead organisms^23,26^. The detection of biodiversity information that originates from a living organism mainly depends on the dispersal of this living organism^11,20^. The detection of biodiversity information that originates from a dead organism mainly depends on its transport capacity and degradation rate^12,22,29^. In summer and autumn, as driven by active organisms, more eDNA was input into the river system. In particular, the surface runoff caused by rain can input more eDNA from terrestrial soil into the river system and can preserve them in soil aggregates^30^. In the present study, the highest proportion of bacteria in riparian soil was detected in riverine water in summer and autumn, and the rain promoted this phenomenon (Fig. 3 and Table 1, Supplementary Table S3). The proportion of effective upstream-to-downstream WBIF was significantly higher in summer and autumn than in spring, as well as being higher on rainy days than on cloudy days (Table 2). eDNA (originated from dead organisms) degrades over time in a logistic manner (a half-life time)^12,22,27,31^, which was described in this study as degrading by half-life distance in a lotic system, which integrates the transport capacity and the degradation rate. In the present work, as driven by runoff discharge and flow velocity (Supplementary Table S1), the half-life distance of noneffective WBIF was significantly farther in the summer than in autumn and in spring (Table 2).

The biodiversity information monitoring effectiveness of riverine water eDNA, as approximated by the transportation effectiveness of WBIF, was impacted by the eDNA degradation rate in WBIF, and there were taxonomy-specific eDNA degradation rates^27^, species-specific eDNA degradation rates^17^, and form-specific eDNA degradation rates^28^. We hypothesized that the monitoring effectiveness of riverine water eDNA would vary with taxonomic communities. In the present case, the results revealed the detection of a significantly higher monitoring effectiveness of riverine water eDNA (both riparian-to-river and downstream-to-upstream) for bacterial communities than for eukaryotic communities (Tables 3, 4). Considering the insufficient read depth on the bacterial community (16S rRNA gene, Supplementary Fig. S2), the detection capacity on bacterial group was already underestimated. A significantly higher monitoring effectiveness of riverine water eDNA was found for micro-eukaryotic communities (fungi) than for overall eukaryotic communities (including micro- and macro-organisms) (Tables 3, 4). This indicates that the monitoring effectiveness varied with different taxonomic communities, and the effectiveness of monitoring eukaryotic communities was significantly lower than for monitoring bacterial communities; in addition, the effectiveness of monitoring macrobe communities was significantly lower than for monitoring microbe communities.

eDNA surveys that are based on metabarcoding can actually acquire information across the taxonomic tree of life^5,6,11,32,33^. However, eDNA that originates from different taxonomic groups has a different probability of being left in the environment and input into water^6,8,9,34^. van Bochove et al. inferred that the eDNA contained inside of cells and mitochondria is especially resilient against degradation (i.e., intracellular vs. extracellular effects)^28^. In the present case, more bacteria than eukaryotes and more microorganisms than macroorganisms (both OTU and species levels) in riparian soil could be detected in riverine water (Table 3). The half-life distance of noneffective WBIF for bacteria (detected by the 16 s RNA gene) was much farther than that for unicellular eukaryotes (detected by the ITS gene, which is mainly unicellular), than that for multicellular eukaryotes (as detected by the CO1 gene, which is mainly multicellular) (Table 4). We inferred that the eDNA contained inside of bacterial cells was more resilient against degradation than that contained inside of unicellular eukaryotic cells (i.e., prokaryotic cells vs. eukaryotic cells), as well as compared to the eDNA contained inside of multicellular eukaryotic cells or extracellular mitochondria (i.e., unicellular eukaryotic cells vs. multicellular eukaryotic cells or extracellular mitochondria).

In previous studies, the effectiveness of using water eDNA to monitor terrestrial organisms was indicated by the detection probability^8,9,34^, and the effectiveness of using downstream water eDNA to monitor upstream organisms was indicated by the detectable distance^7,12,17,19,20,35^. In this study, we approximated the biodiversity information monitoring effectiveness by the WBIF transportation effectiveness and proposed its assessment framework, in which we described the riparian-to-river monitoring effectiveness with the proportion of biodiversity information in riparian soil that was detected by using riverine water eDNA samples. Additionally, we described the downstream-to-upstream monitoring effectiveness with the proportion of biodiversity information in upstream site water eDNA samples that was detected by 1-km downstream site water eDNA samples, and the runoff distance of that 50% of dead bioinformation (i.e., the bioinformation labeling the biological material that lacked life activity and fertility) could be monitored. These indicators provided new usable assessment tools for designing monitoring projects and for evaluating monitoring results.

In the optimal monitoring season and weather condition (a summer rainy day) in the Shaliu river basin on the Qinghai–Tibet Plateau, by using riverine water eDNA, we were able to monitor as much as 87.95% of bacterial species, 76.18% of fungal species, and 53.52% of eukaryotic species from riparian soil, along with as much as 98.69% of bacterial species, 95.71% of fungal species, and 92.41% of eukaryotic species from 1 km upstream (Table 4). The half-life distance of the noneffective WBIF was respectively 17.82 km, 5.96 km, and 5.02 km for bacteria, fungi, and metazoans at the species level (Table 4). When considering the fact that the monitoring effectiveness of eDNA can not only vary with season, weather, and taxonomic communities, but can also vary with rivers and watersheds with different environmental conditions^12,17,19,23^, more studies on the monitoring effectiveness for each taxonomic community in other watersheds with different environmental conditions are needed.

eDNA metabarcoding surveys are relatively cheaper, more efficient, and more accurate than traditional surveys in aquatic systems^10,13^, although this is certainly not true in all circumstances^36^. Sales et al. show that the detection probability of using riverine water eDNA to monitor the semi-aquatic and terrestrial mammals in natural lotic ecosystems in the UK was 40–67%, which provided comparable results to conventional survey methods per unit of survey effort for three species (water vole, field vole and red deer); in other words, the results from 3 to 6 water replicates would be equivalent to the results from 3 to 5 latrine surveys and 5–30 weeks of single camera deployment^9^. In the current case, the riverine water eDNA samples detected 53.52% of eukaryotic species from riparian soil samples. As the bioinformation in WBIF includes the biodiversity information of all taxonomic communities, the information of all taxonomic communities could be monitored by using riverine water eDNA, although variability in monitoring effectiveness exists among different taxonomic communities. We anticipate that, in future biodiversity research, conservation, and management, we will be able to efficiently monitor and assess the aquatic and terrestrial biodiversity by simply using riverine water eDNA samples.

In summary, to test the idea of using riverine water eDNA to simultaneously monitor aquatic and terrestrial biodiversity, we proposed a monitoring effectiveness assessment framework, in which the land-to-river monitoring effectiveness was indicated by detection probability, and the upstream-to-downstream monitoring effectiveness was described by the detection probability per kilometer runoff distance and by the half-life distance of dead bioinformation. In our case study, in the Shaliu River watershed on the Qinghai-Tibet Plateau, and on summer rainy days, 43–76% of species information in riparian sites could be detected in adjacent riverine water eDNA samples, 92–99% of species information from upstream sites could be detected in a 1-km downstream eDNA sample, and the half-life distances of dead bioinformation for bacteria was approximately 13–19 km and was approximately 4–6 km for eukaryotes. The indicators in the assessment framework that describe the monitoring effectiveness provide usable assessment tools for designing monitoring projects and for evaluating monitoring results. In future ecological research, biodiversity conservation, and ecosystem management, riverine water eDNA may be a general diagnostic procedure for routine watershed biodiversity monitoring and assessment.

## Materials and methods

### Study area

The Shaliu River basin (37° 10′–37° 52′ N, 100° 17′–99° 32′ E), as a sub-basin of the Qinghai Lake basin, is located 3196 m above sea level on the Qinghai–Tibet Plateau (Fig. 1). The Shaliu River is 106 km long, with a catchment area of 1320 km^2^. Grassland is the main land cover type, accounting for more than 90% of the watershed area. Less than 5% of the watershed area has been seriously changed by human activity, such as transformation into cultivated land and building land (http://www.gangcha.gov.cn/html/2125/item.html). Due to its simple ecosystem assemblages (only grassland, aquatic ecosystem and building land) and weak disturbance by human activity, the Shaliu River basin is a natural simplified model for investigating the effectiveness of monitoring aquatic and terrestrial biodiversity information using riverine water eDNA.

### Sampling and sequencing

To identify the seasonal variation of monitoring effectiveness, on April 8 and 9, June 25 and 26, and September 19 and 20 of 2019, we collected eDNA samples (spring group, summer group, and autumn group, respectively), including 27 riparian soil eDNA samples and 27 riverine water eDNA samples. The samples were collected from 9 transects (including riverine sampling sites and riparian sampling sites) of the Shaliu River (Fig. 1). The weather and hydrological conditions of each group are summarized in Supplementary Table S1. A 5-mL surface soil sample was collected using a 5-mL sterilized centrifuge tube from the riparian site (5 m from the river) of each transect. A 1.5-L surface water sample was collected using a 1.5-L sterilized bottle (rinsed three times with sampling water) from the riverine site of each transect. Because keeping the samples cool can reduce the rate of eDNA decay and is a convenient and efficient method for conserving eDNA samples^37^, field samples were transported in an ice bath (0 °C) to the laboratory of the Rescue and Rehabilitation Center of Naked Carps of Qinghai Lake. To obtain the eDNA of most taxonomic communities^25,38^, riverine water samples (with purified water used as a negative control) were filtered by using 0.2-μm membrane filters (JinTeng, Tianjin, PRC) to obtain the eDNA sample in the laboratory (with every step following the operation specification of molecular biology experiment to control for contamination and using bleach to wash the experimental apparatus). Subsequently, the filter membranes of each riverine water sample were placed in a 50-mL sterilized centrifuge tube. The samples were transported at − 20 °C (in a dry ice bath), and stored at − 80 °C (in an ultra-low temperature freezer) until DNA extraction. More details are provided in Table 5 and Supplementary Material 1.

**Table 5:** The steps of sampling and sequencing.

Sample types	Riparian soil eDNA sample	Riverine water eDNA sample
Sampling site	Riparian area (5 m distance from the river) of each transect	River of each transect
Step 1: field sampling	Collecting 5 mL riparian soil using a 5-mL sterilized centrifuge tube	Collecting 1.5 L of riverine water using a 1.5-L sterilized bottle (rinsed three times with sampling water)
Step 2: field samples transport	Transporting to the laboratory of the Rescue and Rehabilitation Center of Naked Carps of Qinghai Lake at 0 °C (in an ice bath)	
Step 3: samples pretreatment		Filtering riverine water using 0.2-μm membrane filters and placing the filters of each riverine water sample into a 50-mL sterilized centrifuge tube
Step 4: samples frozen	Freezing the tubes in a − 20 °C refrigerator	
Step 5: samples transport	Transporting the tubes at − 20 °C (in a dry ice bath)	
Step 6: samples store	Storing the tubes at − 80 °C (in an ultra-low temperature freezer) until DNA extraction	
Step 7: DNA extraction	Extracting DNA using an FastDNA SPIN Kit for Soil	
Step 8: DNA quality testing	Determining the final DNA concentration and purity using a NanoDrop 2000 UV–Vis spectrophotometer, checking the DNA quality using 1% agarose gel electrophoresis	
Step 9: PCR amplification—primer (with barcode)	1. Bacterial 16S rRNA gene: 338F (5′-ACTCCTACGGGAGGCAGCAG-3′) 806R (5′-GGACTACHVGGGTWTCTAAT-3′) 2. Fungal ITS gene: ITS1F (5′-CTTGGTCATTTAGAGGAAGTAA) ITS2R (5′-GCTGCGTTCTTCATCGATGC) 3. Eukaryotic mitochondrial CO1 gene: mlCOIintF (5′-GGWACWGGWTGAACWGTWTAYCCYCC) jgHCO2198R (5′-TANACYTCNGGRTGNCCRAARAAYCA)	
Step 9: PCR amplification—reaction system (3 duplicate, with blank controls)	20-μL mixtures containing 4 μL of 5 × FastPfu Buffer, 2 μL of 2.5 mM dNTPs, 0.8 μL of each primer (5 μM), 0.4 μL of FastPfu Polymerase, 0.2 μL of BSA, 10 ng of template DNA and ddH_2_O	
Step 10: PCR amplification—program (GeneAmp 9700, ABI, USA)	1. Bacterial 16S rRNA gene: 3 min of denaturation at 95 °C; 29 cycles of 30 s at 95 °C, 30 s for annealing at 55 °C, and 45 s for elongation at 72 °C; and a final extension at 72 °C for 10 min 2. Fungal ITS gene: 3 min of denaturation at 95 °C; 37 cycles of 30 s at 95 °C, 30 s for annealing at 53 °C, and 45 s for elongation at 72 °C; and a final extension at 72 °C for 10 min 3. Eukaryotic mitochondrial CO1 gene: 5 min of denaturation at 94 °C; 35 cycles of 60 s at 94 °C, 120 s for annealing at 47 °C, and 60 s for elongation at 72 °C; and a final extension at 72 °C for 5 min	
Step 11: PCR product testing	Testing PCR product quality using 2% agarose gel electrophoresis	
Step 12: PCR product extraction and purification	PCR products were extracted from a 2% agarose gel using an AxyPrep DNA Gel Extraction Kit, and then purified using an QIAquick PCR Purification Kit	
Step 13: PCR product quantification	PCR products were quantified using QuantiFluor-ST	
Step 14: Miseq library preparation (TruSeq DNA Sample Prep Kit)	Adding the standard tags of Illumina to PCR products according another PCR program, extracting, purifying and checking tagged PCR products, preparing single-stranded DNA	
Step 15: Miseq sequencing	Purified amplicons were pooled in equimolar amounts and subjected to paired-end sequencing on an Illumina MiSeq platform	
Step 16: raw sequence treatment	Raw fastq files were demultiplexed, quality-filtered by Trimmomatic and merged by FLASH	
Step 17: clustering OTU	Operational taxonomic units (OTUs) were clustered with a 97% similarity cutoff using UPARSE, and chimeric sequences were identified and removed using UCHIME	
Step 18: taxonomy identification	The taxonomies of each sequence were analyzed by the RDP Classifier Bayesian algorithm against the corresponding database using a confidence threshold of 70% Database selection: 1. Bacterial 16S rRNA gene: Silva132/16S_Bacteria database 2. Fungal ITS gene: Unite8.0/ITS_Fungi database 3. Eukaryotic mitochondrial CO1 gene: nt database (standard database)	
Step 18: communities analysis	The OTU numbers, types and taxonomic features of the samples were analyzed. Community Chao richness at the OTU level was examined using the software of Mothur	

To identify the taxonomic variation of monitoring effectiveness, we analyzed three taxonomic communities using the metabarcoding of the 16S rRNA, ITS, and mitochondrial CO1 genes^39–41^. As long DNA fragments show a higher decay rate than short fragments^22^, short fragments better reflect community richness than long fragments^22,31^. We restricted the amplified fragment length to 300–500 bp and selected the primers 338F/806R, ITS1F/ITS2R, and mlCOIintF/jgHCO2198R to detect bacteria, fungi, and metazoan, respectively^39–41^. As the extraction of eDNA^42,43^, amplification approach, and sequencing^44^ can impact the results of eDNA monitoring, a consistent DNA extraction method and amplification approach should be used for comparisons among samples^41,45,46^. Commercial eDNA labs can help^11^, in which all approaches (including eDNA extraction, primer synthesis, amplification approach, sequencing, and contamination control, among others) could be standard. In our work, samples were processed by Shanghai Majorbio Bio-pharm Technology Co., Ltd (Shanghai, China). The details are provided in Table 5 and Supplementary Material 1.

On the free online Majorbio Cloud Platform (www.majorbio.com), we analyzed the raw sequences data, and we obtained the types of operational taxonomic unit (OTU), the sequence number of each OTU, and the taxonomic features of each sample; additionally, we examined the community richness (Chao richness index at the OTU level).

### WBIF analysis

The WBIF (including land-to-river and upstream-to-downstream WBIF) of each group was assessed to reveal the effectiveness of using riverine water eDNA to monitor the biodiversity information in riverine sites and riparian sites. In the current WBIF analysis, all of the statistical analyses used the OTUs and species in each sample. The processing approach was simply described as follows (indicated by the OTU type).

The transportation effectiveness of WBIF was indicated by the proportion of input OTUs (i.e., the common types between the source site sample and the pool site sample) to output OTUs (the total types of source site sample) (Eq. 1).

1 $$e=\frac{\mathrm{Num}\left({S}_{\mathrm{OTU}} \cap {P}_{\mathrm{OTU}}\right)}{\mathrm{Num}\left({S}_{\mathrm{OTU}}\right)},$$

where *e* denotes the transportation effectiveness of WBIF; *S*_*OTU*_ denotes the OTU assemblage of the source site sample (i.e., the adjacent riparian soil eDNA sample in the land-to-river WBIF or the adjacent upstream water eDNA sample in the upstream-to-downstream WBIF); and *P*_*OTU*_ denotes the OTU assemblage of the pool site sample (i.e., the adjacent riverine water eDNA sample in the land-to-river WBIF or the adjacent downstream water eDNA sample in the upstream-to-downstream WBIF).

As the distance of the land-to-river WBIF was less than 5 m in the present case study, the transportation effectiveness of the land-to-river WBIF was assumed to be constructed by transport capacity and environmental filtration (no degradation rate). The transportation effectiveness of the land-to-river WBIF could be indicated by the proportion of the common types shared between adjacent riparian soil eDNA samples and riverine water eDNA samples to the total types of riparian soil eDNA samples (Eq. 1). The transport capacity of the land-to-river WBIF could be indicated by the proportion of the common types shared between adjacent riparian soil eDNA samples and riverine water eDNA samples to the common types shared between the riparian soil eDNA sample and all riverine water eDNA samples in the corresponding group (Eq. 2). The environmental filtration of the land-to-river WBIF could be indicated by the proportion of the types included in the riparian soil eDNA sample, but not in any riverine water eDNA sample to the total types in the riparian soil eDNA sample (Eq. 3).

2 $$t=\frac{\mathrm{Num}\left({S}_{\mathrm{OTU}} \cap {P}_{\mathrm{OTU}}\right)}{\mathrm{Num}\left({S}_{\mathrm{OTU}} \cap {W}_{\mathrm{OTU}}\right)},$$

3 $$f=1-\frac{\mathrm{Num}\left({S}_{\mathrm{OTU}} \cap {W}_{\mathrm{OTU}}\right)}{\mathrm{Num}\left({S}_{\mathrm{OTU}}\right)},$$

where *t* denotes the transport capacity; *f* denotes the environmental filtration; *S*_*OTU*_ denotes the OTU assemblage of the source site sample (i.e., the riparian soil eDNA sample); and *W*_*OTU*_ denotes the OTU assemblage of all riverine water eDNA samples.

WBIF included the effective WBIF (i.e., the flow or migration of living organisms) and noneffective WBIF (i.e., the flow of the bioinformation labeling the biological material that lacked life activity and fertility [dead bioinformation]). The transportation effectiveness of upstream-to-downstream WBIF was determined by the different features of effective WBIF and noneffective WBIF. The effective WBIF was impacted by transport capacity and environmental filtration. The noneffective WBIF was impacted by transport capacity and degradation rate. We established the following presuppositions: (1) the transport capacity was consistent in a defined runoff condition of a definite season and weather condition; (2) the proportion of noneffective WBIF at each site was consistent; (3) the noneffective WBIF degraded over time (i.e., distance) in a logistic manner; and (4) the environmental filtration was consistent in a definite environmental change. These four presuppositions did not exactly describe the factual complex WBIF processes driven by various environmental factors, but they provided a possibility of constructing a model to approximately address the complex WBIF processes. The transportation effectiveness of the upstream-to-downstream WBIF could be described by an equation (Eq. 4), in which the transportation effectiveness was the function of runoff distance, and the transport capacity, environmental filtration, and degradation rate were parameters that could be estimated according to the sets of transportation effectiveness and runoff distance. In practice, as WBIF are impacted by varying factors at any site and time, the analytical solution of the parameters in Eq. (4) is impossible. Therefore, we suggested that Eq. (4) could be programming-solved, according to the evolutionary algorithm in Microsoft Excel. As there were only approximate solutions of the parameters in Eq. (4), we suggested obtaining several sets (such as 30 sets) of approximate solutions, after which a statistical analysis could be performed for each parameter.

4 $$e={t}^{d}\times \left[\left(1-k\right)\times \left(1-f\right)+k\times {\left(\frac{1}{2}\right)}^{\left(\frac{d}{D}\right)}\right],$$

where *e* denotes the transportation effectiveness of WBIF; *t* denotes the transport capacity; *d* denotes the distance of WBIF; *k* denotes the proportion of the noneffective WBIF; *f* denotes the environmental filtration; and *D* denotes the half-life distance of the noneffective WBIF.

## Supplementary Information

Supplementary  methods.

Supplementary  figures & tables.

## Data Availability

The datasets that were generated for this study can be found in the China National GeneBank Sequence Archive (CNSA, https://db.cngb.org/cnsa/) of the China National GeneBank database (CNGBdb) under accession number CNP0001046.
