# Supplementary material for: Effectiveness assessment of using riverine water eDNA to simultaneously monitor the riverine and riparian biodiversity information
Source: Sci Rep. 2021 Dec 20;11:24241. doi: 10.1038/s41598-021-03733-7 (PMC8688430; doi:10.1038/s41598-021-03733-7)
Supplement: Supplementary file 1 — Supplementary methods. [file 41598_2021_3733_MOESM1_ESM.docx]

## Supplementary material 1

### Sampling and sequencing

To assess the monitoring effectiveness (approximately indicated by the watershed biological information flow [WBIF] transportation effectiveness), we compared the biodiversity information detected in adjacent upstream-to-downstream samples and that detected in adjacent riparian-to-river samples. To identify the seasonal variation in monitoring effectiveness, we sampled at three different times in the spring, summer, and autumn. To identify the taxonomic variation of the monitoring effectiveness, we analyzed three taxonomic communities by using metabarcoding of the 16S rRNA gene, ITS gene, and mitochondrial CO1 gene^1-3^. To determine the eDNA of most taxonomic communities, we selected the finest filter membrane (0.2 μm) to filter the riverine water samples^4,5^. Due to the fact that keeping the samples cooled can reduce the rate of eDNA decay and is a convenient and efficient method for conserving eDNA samples^6^, we maintained our eDNA samples in an ice bath or in a dry ice bath when they were transported; additionally, we maintained them in an ultralow temperature freezer for storage.

**Field sampling**

On April 8 and 9, June 25 and 26, and September 19 and 20 of 2019, we collected eDNA samples (spring group, summer group, and autumn group, respectively), including 27 riparian soil eDNA samples and 27 riverine water eDNA samples. The samples were collected from 9 transects (including riverine sampling sites and riparian sampling sites) of the Shaliu River (Fig. 1). A 5-mL soil eDNA sample (actual surface soil) was collected by using a 5-mL sterilized centrifuge tube from the riparian site (located 5 m from the river) of each transect and transported in an ice bath to the laboratory of the Rescue and Rehabilitation Center of Naked Carps of Qinghai Lake at 0°C. Subsequently, the tubes with riparian soil eDNA samples were frozen in a -20°C refrigerator, transported at -20°C (in a dry ice bath), and stored at -80°C (in an ultralow temperature freezer) until DNA extraction was performed. A 1.5-L riverine surface water sample (actual riverine water) was collected by using a 1.5-L sterilized bottle (rinsed three times with sampling water) from the river site of each transect and transported at 0°C (in an ice bath) to the laboratory. Afterwards, riverine water samples (with purified water used as a negative control) were filtered by using 0.2-μm membrane filters (JinTeng, Tianjin, PRC) to obtain the eDNA sample in the laboratory (with every step following the operation specification of molecular biology experiment to control for contamination and using bleach to wash the experimental apparatus), and the membrane filters of each riverine water eDNA sample were placed in a 50-mL sterilized centrifuge tube. The tubes with the membrane filters of riverine water eDNA samples were then frozen in a -20°C refrigerator, transported at -20°C (in a dry ice bath), and stored at -80°C (in an ultralow temperature freezer) until DNA extraction was performed.

In the first sampling period (the spring group), during April 8 and 9, the air temperature was -6-8°C, the water temperature was -0.5-0.7°C, the frozen river was beginning to thaw, the runoff volume was 1.8-3.9 m³/s, the flow velocity was 0.63-1.04 m/s, the riparian soil was still frozen, and both days were cloudy with freezing, heavy winds. On the frozen days of April 8 and 9, the river was clear; on these days, we sampled 18 samples (9 riverine water samples and 9 riparian soil samples) at 9 transects by following the downstream-to-upstream direction.

In the second sampling period (the summer group), during June 25 and 26, the air temperature was 7-17°C, the water temperature was 4.3-16.4°C, the runoff volume was 29.9-45.5 m³/s, and the flow velocity was 0.84-2.03 m/s. On the sunny day of June 25, the river was clear; on this day, we collected 4 samples (2 riverine water samples and 2 riparian soil samples) from the SL1 and SL2 transects (two downstream transects). It began to rain (a light rain, less than 10 mm) on the night of June 25; on the rainy day (a light rain, less than 10 mm) of June 26, the river was turbid (different transects, different turbidities); we collected 14 samples (7 riverine water samples and 7 riparian soil samples) from the last 7 transects by following the downstream-to-upstream direction.

In the third sampling period (the autumn group) on September 19 and 20, the air temperature was 0-10°C, the water temperature was 0.2-8.8°C, part of the transects started to freeze, the runoff volume was 5.7-12.8 m³/s, and the flow velocity was 0.57-0.88 m/s. On the rainy day (a light rain, less than 10 mm) of September 19, the downstream river section was turbid, and we collected 4 samples (2 riverine water samples and 2 riparian soil samples) from the SL1 and SL2 transects (two downstream transects). On the cloudy day of September 20, the river was clear, and we collected 14 samples (7 riverine water samples and 7 riparian soil samples) from the last 7 transects by following the downstream-to-upstream direction.

**DNA Extraction and Sequence Analysis**

As the extraction of eDNA^7,8^, metabarcoding selection^1-3^, amplification approach, and sequencing^9^ can impact the results of eDNA monitoring, consistent methods should be used for comparisons among the samples^3,10,11^. Commercial eDNA labs can help with consistency^12^, in which all of the approaches (including eDNA extraction, primer synthesis, amplification approach, sequencing, and contamination control) could be standardized. We sent the samples to these labs and selected the primers, and they provided us with the results, which included a set of sequences. Additionally, they provided an interactive platform to analyze the sequences.

In our study, our samples were processed by Shanghai Majorbio Bio-pharm Technology Co., Ltd. (Shanghai, China), and our data were analyzed on the free online platform of the Majorbio Cloud Platform (www.majorbio.com). As long DNA fragments exhibit a higher decay rate than short fragments^13,14^, short fragments better reflect community richness than long fragments^15,16^. Thus, we restricted the amplified fragment length to 300-500 bp. To distinguish the taxonomic communities, we selected the primers 338F/ 806R, ITS1F/ ITS2R, and mlCOIintF/ jgHCO2198R to indicate bacteria, fungi, and eukaryotes, respectively^1-3^.

DNA was extracted from the eDNA samples (with the use of a negative control) by using the FastDNA SPIN Kit for Soil and the FastPrep Instrument (MP Biomedicals, Santa Ana, CA), according to the manufacturer’s protocols in the eDNA-specific lab. Subsequently, the final DNA concentration and purity were determined by using a NanoDrop 2000 UV-vis spectrophotometer (Thermo Scientific, Wilmington, USA), and DNA quality was verified via 1% agarose gel electrophoresis.

Three sets of specific primers with barcodes (338F/ 806R, ITS1F/ ITS2R, and mlCOIintF/ jgHCO2198R) were synthesized. The bacterial 16S rRNA gene was amplified with the primers 338F (5’-ACTCCTACGGGAGGCAGCAG-3’) and 806R (5’-GGACTACHVGGGTWTCTAAT-3’) by using a PCR thermocycler system (GeneAmp 9700, ABI, USA) (with blank controls) and the following program: 3 min of denaturation at 95°C; 29 cycles of 30 s at 95°C, 30 s for annealing at 55°C, and 45 s for elongation at 72°C; and a final extension at 72°C for 10 min. The PCR assays were performed in triplicate 20-μL mixtures containing 4 μL of 5× FastPfu Buffer, 2 μL of 2.5 mM dNTPs, 0.8 μL of each primer (5 μM), 0.4 μL of FastPfu Polymerase, 0.2 μL of BSA, and 10 ng of template DNA. The fungal ITS gene was amplified with the primers ITS1F (5’-CTTGGTCATTTAGAGGAAGTAA) and ITS2R (5’-GCTGCGTTCTTCATCGATGC) by using a PCR thermocycler system (GeneAmp 9700, ABI, USA) (with blank controls) and the following program: 3 min of denaturation at 95°C; 37 cycles of 30 s at 95°C, 30 s for annealing at 53°C, and 45 s for elongation at 72°C; and a final extension at 72°C for 10 min. The PCR assays were performed in triplicate 20-μL mixtures containing 4 μL of 5× FastPfu Buffer, 2 μL of 2.5 mM dNTPs, 0.8 μL of each primer (5 μM), 0.4 μL of FastPfu Polymerase, 0.2 μL of BSA, and 10 ng of template DNA. The eukaryotic mitochondrial CO1 gene was amplified with the primers mlCOIintF (5’-GGWACWGGWTGAACWGTWTAYCCYCC) and jgHCO2198R (5’-TANACYTCNGGRTGNCCRAARAAYCA) by using a PCR thermocycler system (GeneAmp 9700, ABI, USA) (with blank controls) and the following program: 5 min of denaturation at 94°C; 35 cycles of 60 s at 94°C, 120 s for annealing at 47°C, and 60 s for elongation at 72°C; and a final extension at 72°C for 5 min. The PCR assays were performed in triplicate 20-μL mixtures containing 4 μL of 5× FastPfu Buffer, 2 μL of 2.5 mM dNTPs, 0.8 μL of each primer (5 μM), 0.4 μL of FastPfu Polymerase, 0.2 μL of BSA, and 10 ng of template DNA. The PCR products were extracted and further purified by using the AxyPrep DNA Gel Extraction Kit (Axygen Biosciences, Union City, CA, USA). The resulting PCR products of the same sample were mixed together and verified via 2% agarose gel electrophoresis.

The PCR product amplicons were quantified by using QuantiFluor -ST (Promega, U.S.), according to the manufacturer’s protocol. According to the sequencing requirements of each sample, the PCR products were mixed on the basis of the proportions. The standard adaptors provided by Illumina (Illumina, San Diego, USA) were linked to the sequencing regions according to the PCRs. Adapter dimers were removed by using beads. Furthermore, single-stranded DNA fragments were generated by using sodium hydroxide. Sample libraries were pooled in equimolar amounts and subjected to paired-end sequencing on an Illumina MiSeq platform (Illumina, San Diego, USA), according to standard protocols.

Raw fastq files were demultiplexed, quality-filtered by using Trimmomatic, and merged by using FLASH (https://ccb.jhu.edu/software/FLASH/index.shtml). Operational taxonomic units (OTUs) were clustered with a 97% similarity cutoff by using UPARSE (http://www.drive5.com/uparse/), and chimeric sequences were identified and removed by using UCHIME (http://www.drive5.com/uchime/). The taxonomies of each 16S rRNA, ITS, and CO1 gene sequence were analyzed by using the RDP Classifier Bayesian algorithm (http://sourceforge.net/projects/rdp-classifier/) against the Silva132/16S_Bacteria database (http://www.arb-silva.de) with the use of a confidence threshold of 70%, against the Unite8.0/ITS_Fungi database (http://unite.ut.ee/index.php) with the use of a confidence threshold of 70%, and against the nt database (standard database), respectively. Additionally, the OTU numbers, types, and taxonomic features of the samples were analyzed. Community richness (Chao richness index at the OTU level) was examined to determine the variation among the three groups by using the Mothur software (https://www.mothur.org/wiki/Download_mothur). The data were analyzed online by using the Majorbio Cloud Platform (www.majorbio.com).

Additionally, the raw data were deposited in the China National GeneBank Sequence Archive (CNSA, https://db.cngb.org/cnsa/) of the China National GeneBank database (CNGBdb) under accession number CNP0001046.

### WBIF Analysis

The WBIF (watershed biological information flow, including land-to-river and upstream-to-downstream WBIF) of each group was assessed to reveal the effectiveness of using riverine water eDNA to monitor the biodiversity information in riverine sites and riparian sites. In the current WBIF analysis, all of the statistical analyses used the OTUs and species in each sample. The processing approach was described as follows (as indicated by the OTUs).

The transportation effectiveness of the land-to-river and upstream-to-downstream WBIF could be estimated by comparing the OTU assemblages between the adjacent riparian soil eDNA sample and the riverine water eDNA sample and by comparing the OTU assemblages between adjacent (upstream-to-downstream) riverine water eDNA samples. The transportation effectiveness of the WBIF was indicated by the proportion of input OTUs (i.e., the common types between the source site sample and the pool site sample) to output OTUs (the total types of the source site sample) (Eq. 1).

$e=\frac{\mathrm{Num}\left( S_{\mathrm{OTU}} \cap P_{\mathrm{OTU}} \right)}{\mathrm{Num}\left( S_{\mathrm{OTU}} \right)}$ (Eq. 1)

where *e* denotes the transportation effectiveness of the WBIF; *S_OTU_* denotes the OTU assemblage of the source site sample (i.e., the adjacent riparian soil eDNA sample in the land-to-river WBIF or the adjacent upstream water eDNA sample in the upstream-to-downstream WBIF); and *P_OTU_* denotes the OTU assemblage of the pool site sample (i.e., the adjacent riverine water eDNA sample in the land-to-river WBIF or the adjacent downstream water eDNA sample in the upstream-to-downstream WBIF).

As the transportation effectiveness of the WBIF relied on the transport capacity, degradation rate, and environmental filtration, as well as the fact that the distance of the land-to-river WBIF was less than 5 m in the present case study, the transportation effectiveness of the land-to-river WBIF was assumed to be constructed by the transport capacity and environmental filtration (no degradation rate). The transportation effectiveness of the land-to-river WBIF could be indicated by the proportion of the common types that were shared between adjacent riparian soil eDNA samples and riverine water eDNA samples to the total types of riparian soil eDNA samples (Eq. 1). The transport capacity of the land-to-river WBIF could be indicated by the proportion of the common types that were shared between adjacent riparian soil eDNA samples and riverine water eDNA samples to the common types shared between the riparian soil eDNA sample and all of the riverine water eDNA samples in the corresponding group (Eq. 2). The environmental filtration of the land-to-river WBIF could be indicated by the proportion of the types that were included in the riparian soil eDNA sample (but not in any riverine water eDNA sample) to the total types in the riparian soil eDNA sample (Eq. 3).

$t=\frac{\mathrm{Num}\left( S_{\mathrm{OTU}} \cap P_{\mathrm{OTU}} \right)}{\mathrm{Num}\left( S_{\mathrm{OTU}} \cap W_{\mathrm{OTU}} \right)}$ (Eq. 2)

$f=1-\frac{\mathrm{Num}\left( S_{\mathrm{OTU}} \cap W_{\mathrm{OTU}} \right)}{\mathrm{Num}\left( S_{\mathrm{OTU}} \right)}$ (Eq. 3)

where *t* denotes the transport capacity; *f* denotes the environmental filtration; *S_OTU_* denotes the OTU assemblage of the source site sample (i.e., the riparian soil eDNA sample); and *W_OTU_* denotes the OTU assemblage of all of the riverine water eDNA samples.

The WBIF includes the effective WBIF (i.e., the flow or migration of living organisms) and the noneffective WBIF (i.e., the flow of bioinformation labeling biological material that lacks life activity and fertility [dead bioinformation]). The transportation effectiveness of the upstream-to-downstream WBIF was determined by the different features of the effective WBIF and the noneffective WBIF. The effective WBIF was impacted by the transport capacity and environmental filtration. The noneffective WBIF was impacted by the transport capacity and degradation rate. We established the following presuppositions: (1) the transport capacity was consistent in a defined runoff condition of a definite season and definite weather conditions; (2) the proportion of noneffective WBIF at each site was consistent; (3) the noneffective WBIF degraded over time (i.e., over distance) in a logistic manner; and (4) the environmental filtration was consistent in a definite environmental change. These four presuppositions did not exactly describe the complex facts, but they provided the possibility of constructing a model to approximately address the complex facts. The transportation effectiveness of the upstream-to-downstream WBIF could be constructed by the transport capacity of the WBIF, the environmental filtration of the effective WBIF, and the degradation rate of the noneffective WBIF. It could be described by an equation (Eq. 4), in which the transportation effectiveness was a function of runoff distance, and the transport capacity, environmental filtration, and degradation rate were parameters that could be estimated according to the sets of transportation effectiveness and runoff distance. In practice, as the WBIF is impacted by various influencing factors at any site and time, the analytical solution of the parameters in Eq. 4 is impossible. Therefore, we suggested that Eq. 4 could be programming-solved, according to the evolutionary algorithm in Microsoft Excel. As there were only approximate solutions of the parameters in Eq. 4, we suggested obtaining several sets (such as 30 sets) of approximate solutions, after which we would perform statistical analyses for each parameter.

$e=t^{d}\times\left[ \left( 1-k \right)\times\left( 1-f \right)+k\times\left( \frac{1}{2} \right)^{\left( \frac{d}{D} \right)} \right]$ (Eq. 4)

where *e* denotes the transportation effectiveness of the WBIF; *t* denotes the transport capacity; *d* denotes the distance of the WBIF; *k* denotes the proportion of the noneffective WBIF; *f* denotes the environmental filtration; and *D* denotes the half-life distance.

In the present case study, we first sequenced the bacterial 16S rRNA gene of the eDNA samples from three seasonal groups and assessed the transportation effectiveness of the WBIF (as indicated by microbial OTUs) in three seasons. Second, we selected the eDNA samples of the seasonal group that showed the highest WBIF transportation effectiveness, sequenced their fungal ITS gene and eukaryotic mitochondrial CO1 gene, and assessed the WBIF transportation effectiveness indicated by three taxonomic communities (bacteria, fungi, and metazoans) at both the OTU level and species level.

### References

^1^ Wangensteen, O. S., Palacín, C., Guardiola, M. & Turon, X., DNA metabarcoding of littoral hard-bottom communities: high diversity and database gaps revealed by two molecular markers. *PEERJ* **6** e4705 (2018).

^2^ Heeger, F., Wurzbacher, C., Bourne, E. C., Mazzoni, C. J. & Monaghan, M. T., Combining the 5.8S and ITS2 to improve classification of fungi. *METHODS ECOL EVOL* **10** 1702 (2019).

^3^ Giebner, H. *et al.*, Comparing diversity levels in environmental samples: DNA sequence capture and metabarcoding approaches using 18S and COI genes. *MOL ECOL RESOUR* **20** 1333 (2020).

^4^ Eichmiller, J. J., Best, S. E. & Sorensen, P. W., Effects of Temperature and Trophic State on Degradation of Environmental DNA in Lake Water. *ENVIRON SCI TECHNOL* **50** 1859 (2016).

^5^ Li, J., Lawson Handley, L., Read, D. S. & Hänfling, B., The effect of filtration method on the efficiency of environmental DNA capture and quantification via metabarcoding. *MOL ECOL RESOUR* **18** 1102 (2018).

^6^ Sales, N. G., Wangensteen, O. S., Carvalho, D. C. & Mariani, S., Influence of preservation methods, sample medium and sampling time on eDNA recovery in a neotropical river. *Environmental DNA* **1** 119 (2019).

^7^ Hermans, S. M., Buckley, H. L. & Lear, G., Optimal extraction methods for the simultaneous analysis of DNA from diverse organisms and sample types. *MOL ECOL RESOUR* **18** 557 (2018).

^8^ Armbrecht, L. *et al.*, An optimized method for the extraction of ancient eukaryote DNA from marine sediments. *MOL ECOL RESOUR* **20** 906 (2020).

^9^ Nichols, R. V. *et al.*, Minimizing polymerase biases in metabarcoding. *MOL ECOL RESOUR* **18** 927 (2018).

^10^ Nicholson, A. *et al.*, An analysis of metadata reporting in freshwater environmental DNA research calls for the development of best practice guidelines. *Environmental DNA* **2** 343 (2020).

^11^ Dopheide, A., Xie, D., Buckley, T. R., Drummond, A. J. & Newcomb, R. D., Impacts of DNA extraction and PCR on DNA metabarcoding estimates of soil biodiversity. *METHODS ECOL EVOL* **10** 120 (2019).

^12^ Ravindran, S., Turning discarded DNA into ecology gold. *NATURE* **570** 543 (2019).

^13^ Jo, T. *et al.*, Rapid degradation of longer DNA fragments enables the improved estimation of distribution and biomass using environmentalDNA. *MOL ECOL RESOUR* **17** e25 (2017).

^14^ Shogren, A. J. *et al.*, Water Flow and Biofilm Cover Influence Environmental DNA Detection in Recirculating Streams. *ENVIRON SCI TECHNOL* **52** 8530 (2018).

^15^ Jo, T., Arimoto, M., Murakami, H., Masuda, R. & Minamoto, T., Particle size distribution of environmental DNA from the nuclei of marine fish. *ENVIRON SCI TECHNOL* **53** 9947 (2019).

^16^ Wei, N., Nakajima, F. & Tobino, T., A microcosm study of surface sediment environmental DNA: decay observation, abundance estimation, and fragment length comparison. *ENVIRON SCI TECHNOL* **52** 12428 (2018).
