# Supplementary material for: Effectiveness assessment of using riverine water eDNA to simultaneously monitor the riverine and riparian biodiversity information
Source: Sci Rep. 2021 Dec 20;11:24241. doi: 10.1038/s41598-021-03733-7 (PMC8688430; doi:10.1038/s41598-021-03733-7)
Supplement: Supplementary file 2 — Supplementary figures & tables. [file 41598_2021_3733_MOESM2_ESM.docx]

## Supplementary material 2

### Figures (with captions)


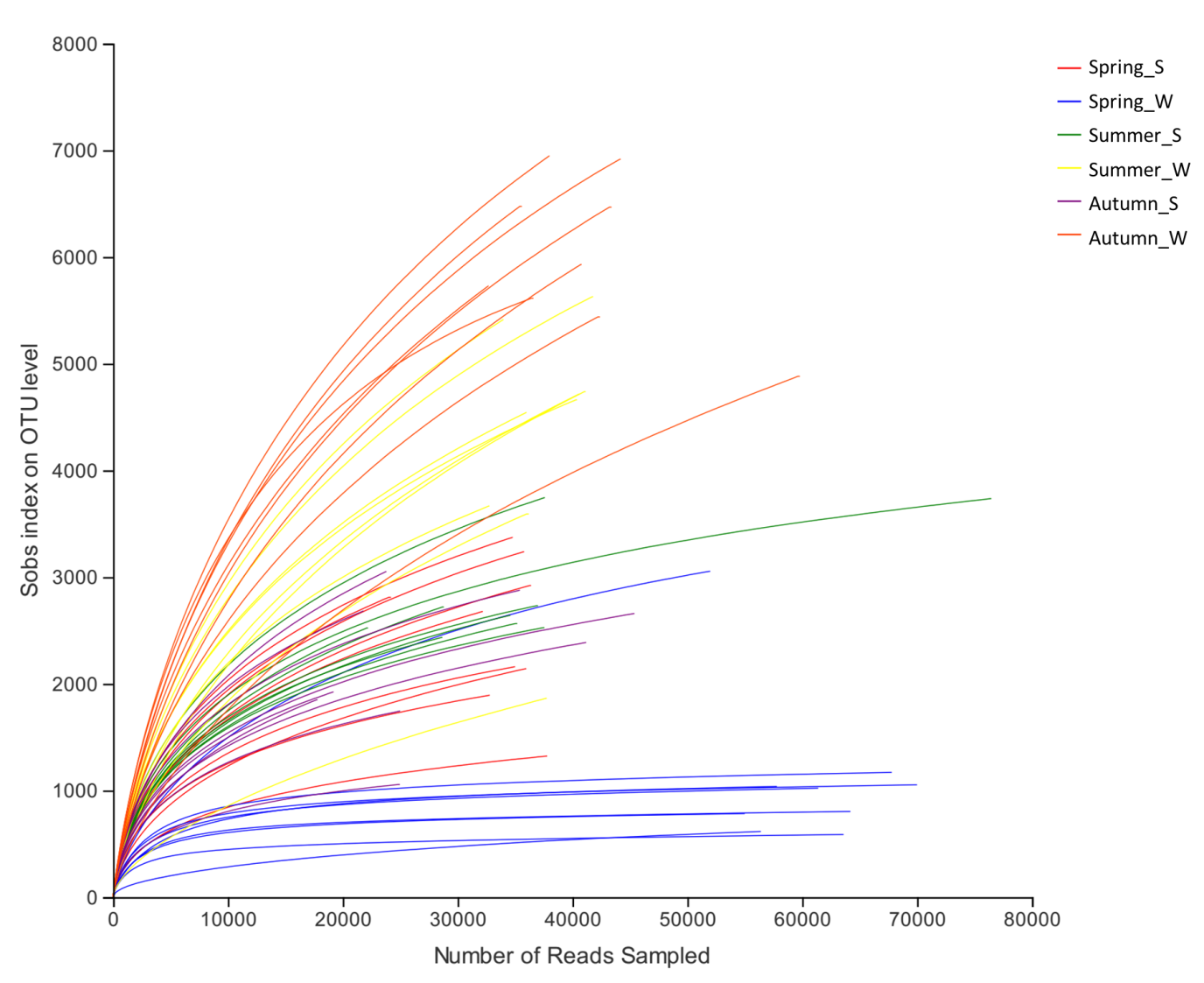


**Figure S1 Rarefaction curves of all sample that were sequenced using the bacterial 16S rRNA gene.**

Spring_S denotes the riparian soil eDNA samples that were sampled during April 2019; Spring_W denotes the riverine water eDNA samples that were sampled during April 2019; Summer_S denotes the riparian soil eDNA samples that were sampled during June 2019; Summer _W denotes the riverine water eDNA samples that were sampled during June 2019; Autumn_S denotes the riparian soil eDNA samples that were sampled during September 2019; Autumn_W denotes the riverine water eDNA samples that were sampled during September 2019. This figure is produced on the Majorbio Cloud Platform ([www.majorbio.com](http://www.majorbio.com)) using Mothur (version v.1.30.2 <https://mothur.org/wiki/calculators/>). Majorbio Cloud Platform is authorized to use the related softwares, such as Flash, UPARSE, UCHIME, RDP Classifier Bayesian algorithm, Mothur and so on.


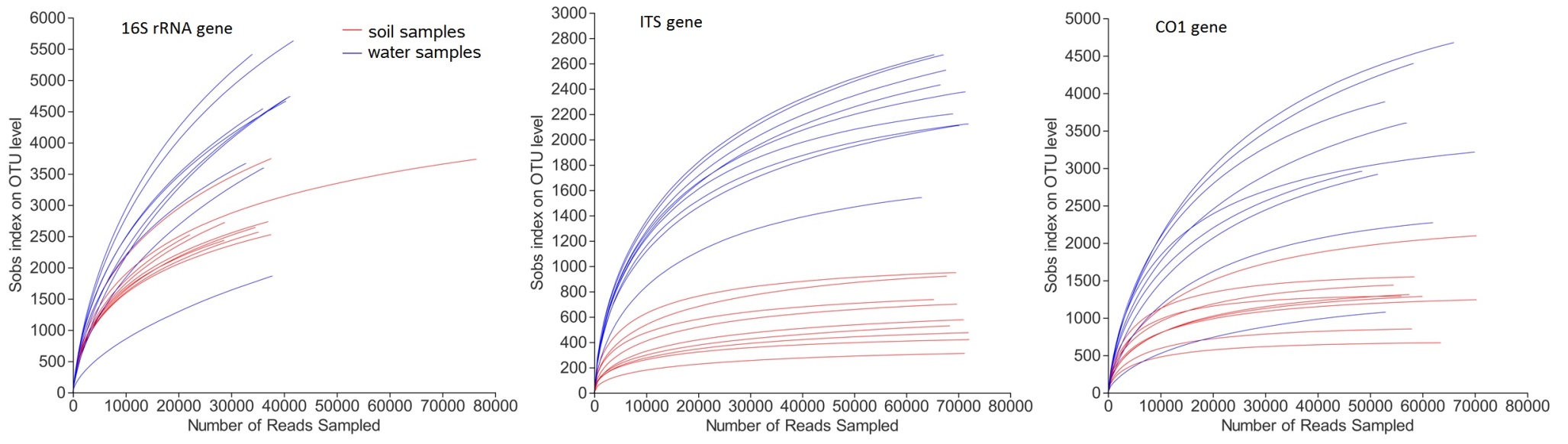


**Figure S2 Rarefaction curves of the samples that were sampled during June 2019 and sequenced by the bacterial 16S rRNA gene, the fungal ITS gene and the eukaryotic mitochondrial CO1 gene.**

These figures are produced on the Majorbio Cloud Platform ([www.majorbio.com](http://www.majorbio.com)) using Mothur (version v.1.30.2 <https://mothur.org/wiki/calculators/>). Majorbio Cloud Platform is authorized to use the related softwares, such as Flash, UPARSE, UCHIME, RDP Classifier Bayesian algorithm, Mothur and so on.

### Tables (with captions)

**Table S1 The weather and hydrological conditions at each sampling time**

|  | Sampling time | Air temperature | Water temperature | Discharge | Flow velocity | Weather conditions |
| --- | --- | --- | --- | --- | --- | --- |
| Spring group | 2019.4.8-9 | -6-8°C | -0.5-0.7°C | 1.8-3.9 m³/s | 0.63-1.04 m/s | frozen days (†)  frozen days (‡) |
| Summer group | 2019.6.25-26 | 7-17°C | 4.3-16.4°C | 29.9-45.5 m³/s | 0.84-2.03 m/s | sunny day (†)  light rain day (‡) |
| Autumn group | 2019.9.19-20 | 0-10°C | 0.2-8.8°C | 5.7-12.8 m³/s | 0.57-0.88 m/s | light rain day (†)  cloudy day (‡) |

(†) Collecting 4 samples (2 riverine water samples and 2 riparian soil samples) from transects of SL1 and SL2 (two downstream transects) along the downstream-to-upstream direction.

(‡) Collecting 14 samples (7 riverine water samples and 7 riparian soil samples) from transects of SL3, SL4, SL4b, SL5m, SL5, SL6 and SL6b (seven upstream transects) along the downstream-to-upstream direction.

**Table S2 Biological information features of the samples of three seasonal groups indicated by the bacterial 16S rRNA gene**

| **Seasonal group** | **Clean sequences** | **Average lengths of sequences (bp)** | **OTUs** | **Biological information features** |
| --- | --- | --- | --- | --- |
| **Spring group** | 1,030,826 | 447.54 (†) | 10,602 | 58 phyla, 141 classes, 424 orders, 782 families, 1,895 genera and 4,537 species |
| **Summer group** | 968,122 | 416.06 | 13,766 |  |
| **Autumn group** | 842,317 | 416.32 | 16,500 |  |

The spring group was sampled during April 2019; the summer group was sampled during June 2019; the autumn group was sampled during September 2019.

(†) As the sequences of spring group samples is our first group sequence results, in analyzing process, the sequences include part of the primers sequences.

**Table S3 Transport capacity, environmental filtration, and transportation effectiveness of watershed biological information flow (WBIF) from the riparian sampling site to the adjacent riverine water sampling site in each sampling transect in three seasons**

|  | **Spring group** | | | **Summer group** | | | **Autumn group** | | |
| --- | --- | --- | --- | --- | --- | --- | --- | --- | --- |
|  | **Transport capacity** | **Environmental filtration** | **Transportation effectiveness** | **Transport capacity** | **Environmental filtration** | **Transportation effectiveness** | **Transport capacity** | **Environmental filtration** | **Transportation effectiveness** |
| S-W_SL6b | 0.099499 | 0.414501 | 0.058256 | 0.504529 | 0.064307 | 0.472084 | 0.566649 | 0.138540 | 0.488145 |
| S-W_SL6 | 0.859828 | 0.383092 | 0.530435 | 0.756467 | 0.048747 | 0.719591 | 0.655770 | 0.153814 | 0.554904 |
| S-W_SL5m | 0.221932 | 0.311564 | 0.152786 | 0.776533 | 0.080882 | 0.713725 | 0.475058 | 0.199631 | 0.380221 |
| S-W_SL5 | 0.226740 | 0.360500 | 0.145000 | 0.655574 | 0.109190 | 0.583992 | 0.597921 | 0.254486 | 0.445759 |
| S-W_SL4b | 0.175403 | 0.401328 | 0.105009 | 0.667238 | 0.107198 | 0.595712 | 0.539223 | 0.121664 | 0.473619 |
| S-W_SL4 | 0.184110 | 0.388603 | 0.112564 | 0.784735 | 0.080409 | 0.721635 | 0.570324 | 0.120697 | 0.501487 |
| S-W_SL3 | 0.205840 | 0.409836 | 0.121480 | 0.649059 | 0.095981 | 0.586761 | 0.610110 | 0.143771 | 0.522394 |
| S-W_SL2 | 0.176972 | 0.414116 | 0.103685 | 0.568211 | 0.222821 | 0.441602 | 0.758528 | 0.269508 | 0.554098 |
| S-W_SL1 | 0.428305 | 0.503238 | 0.212766 | 0.257329 | 0.216037 | 0.201736 | 0.579158 | 0.470276 | 0.306794 |

S-W denotes the WBIF from the riparian soil eDNA sample (riparian zone) to the riverine water eDNA sample (river). SL1, SL2, SL3, SL4, SL4b, SL5m, SL5, SL6, and SL6b denote the sampling transects. S-W_SL1 denotes the WBIF from the riparian zone to the river at SL1. The spring group, summer group, and autumn group indicate the groups that were sampled in April, June, and September 2019, respectively. Transport capacity indicates the proportion of riparian soil microbes that were transported into the river. Environmental filtration indicates the proportion of riparian soil microbes that could not be kept alive in rivers. Transportation effectiveness indicates the proportion of riparian soil microbes that could be detected in rivers.

**Table S4 Accumulative runoff distance and accumulative transportation effectiveness from the first sampling transect in each chain of the upstream-to-downstream watershed biological information flow (WBIF) in three seasons**

| **Transport Chain** | **Cumulative**  **Distance/km** | **Cumulative Effectiveness** | | |
| --- | --- | --- | --- | --- |
|  |  | **Spring group** | **Summer group** | **Autumn group** |
| Chain A |  |  |  |  |
| SL6b-SL5 | 9 | 0.521352 | 0.798283 | 0.598885 |
| ##-SL4 | 24.5 | 0.412811 | 0.534830 | 0.471711 |
| ##-SL3 | 48 | 0.368327 | 0.486959 | 0.415639 |
| ##-SL2 | 63 | 0.330961 | 0.383295 | 0.308563 |
| ##-SL1 | 70 | 0.119217 | 0.197425 | 0.257389 |
| Chain B |  |  |  |  |
| SL6-SL5 | 8 | 0.217131 | 0.762412 | 0.605484 |
| ##-SL4 | 23.5 | 0.111554 | 0.590463 | 0.469171 |
| ##-SL3 | 47 | 0.093625 | 0.517251 | 0.408623 |
| ##-SL2 | 62 | 0.067231 | 0.414025 | 0.299731 |
| ##-SL1 | 69 | 0.032371 | 0.192146 | 0.248692 |
| Chain C |  |  |  |  |
| SL5m-SL4 | 8.5 | 0.430074 | 0.765880 | 0.667929 |
| ##-SL3 | 32 | 0.334385 | 0.579820 | 0.540136 |
| ##-SL2 | 47 | 0.228181 | 0.448004 | 0.361037 |
| ##-SL1 | 54 | 0.072555 | 0.196740 | 0.298750 |
| Chain D |  |  |  |  |
| SL4b-SL3 | 23 | 0.521333 | 0.738029 | 0.719376 |
| ##-SL2 | 38 | 0.352000 | 0.555864 | 0.437211 |
| ##-SL1 | 45 | 0.108000 | 0.237335 | 0.335324 |

Chains A, B, C, and D denote the 4 chains of the upstream-to-downstream WBIF. In Chain A, SL6b-SL5 denotes the WBIF from sampling transects SL6b to SL5; ##-SL4 denotes the WBIF from sampling transects SL6b to SL5, and then to SL4; ##-SL3 denotes the WBIF from sampling transects SL6b to SL5, SL4, and then to SL3; ##-SL2 denotes the WBIF from sampling transects SL6b to SL5, SL4, SL3, and then to SL2; ##-SL1 denotes the WBIF from sampling transects SL6b to SL5, SL4, SL3, SL2, and then to SL1 (estuary). The spring group, summer group, and autumn group indicate the groups that were sampled in April, June, and September 2019, respectively.

**Table S5 Biological information features of the samples of three taxonomic groups sampled on summer rainy days**

| **Taxonomic group (gene)** | **Clean sequences** | **Average lengths of sequences (bp)** | **OTUs** | **Biological information features** |
| --- | --- | --- | --- | --- |
| 16S rRNA gene | 968,122 | 416.06 | 13,766 | 51 phyla, 128 classes, 380 orders, 687 families, 1,512 genera, 3,532 species |
| ITS gene | 1,287,852 | 237.87 | 7,098 | 15 phyla, 53 classes, 137 orders, 301 families, 644 genera, 1,032 species |
| CO1 gene | 1,283,110 | 317.02 | 17,316 | 43 phyla, 140 classes, 492 orders, 1765 families, 4113 genera, 6836 species |

The 16S rRNA gene, ITS gene, and CO1 gene indicate the taxonomic groups detected by the 16S rRNA gene, ITS gene, and CO1 gene, respectively.

**Table S6 Transport capacity, environmental filtration, and transportation effectiveness of watershed biological information flow (WBIF) from the riparian sampling site to adjacent riverine water sampling site in each sampling transect on summer rainy days, estimated at the OTU level**

|  | **Bacteria (detected by the 16S rRNA gene)** | | | **Fungi (detected by the ITS gene)** | | | **Eukaryotes (detected by the CO1 gene)** | | |
| --- | --- | --- | --- | --- | --- | --- | --- | --- | --- |
|  | **Transport capacity** | **Environmental filtration** | **Transportation effectiveness** | **Transport capacity** | **Environmental filtration** | **Transportation effectiveness** | **Transport capacity** | **Environmental filtration** | **Transportation effectiveness** |
| **S-W_SL6b** | 0.064307 | 0.064307 | 0.472084 | 0.478743 | 0.227143 | 0.370000 | 0.280449 | 0.424885 | 0.161290 |
| **S-W_SL6** | 0.048747 | 0.048747 | 0.719591 | 0.708333 | 0.168111 | 0.589255 | 0.484490 | 0.417885 | 0.282029 |
| **S-W_SL5** | 0.109190 | 0.109190 | 0.583992 | 0.511765 | 0.261672 | 0.377850 | 0.237911 | 0.508555 | 0.116920 |
| **S-W_SL5m** | 0.080882 | 0.080882 | 0.713725 | 0.718750 | 0.274102 | 0.521739 | 0.520629 | 0.517992 | 0.250947 |
| **S-W_SL4** | 0.080409 | 0.080409 | 0.721635 | 0.683196 | 0.234984 | 0.522655 | 0.603175 | 0.417745 | 0.351201 |
| **S-W_SL4b** | 0.107198 | 0.107198 | 0.595712 | 0.637168 | 0.286316 | 0.454737 | 0.436923 | 0.530347 | 0.205202 |
| **S-W_SL3** | 0.095981 | 0.095981 | 0.586761 | 0.467337 | 0.360129 | 0.299035 | 0.522523 | 0.584270 | 0.217228 |
| **S-W_SL2** | 0.222821 | 0.222821 | 0.441602 | 0.756522 | 0.375000 | 0.472826 | 0.425620 | 0.754813 | 0.104357 |
| **S-W_SL1** | 0.216037 | 0.216037 | 0.201736 | 0.519531 | 0.390476 | 0.316667 | 0.171348 | 0.692573 | 0.052677 |

S-W denotes the WBIF from the riparian soil eDNA sample (riparian zone) to the riverine water eDNA sample (river). SL1, SL2, SL3, SL4, SL4b, SL5m, SL5, SL6, and SL6b denote the sampling transects. S-W_SL1 denotes the WBIF from the riparian zone to the river at SL1. Bacteria (detected by the 16S rRNA gene), fungi (detected by the ITS gene), and eukaryotes (detected by the CO1 gene) indicate the groups of bacteria (detected by the 16S rRNA gene), fungi (detected by the ITS gene), and eukaryotes (detected by the CO1 gene), respectively. Transport capacity indicates the proportion of riparian soil microbes that were transported into the river. Environmental filtration indicates the proportion of riparian soil microbes that could not be kept alive in rivers. Transportation effectiveness indicates the proportion of riparian soil microbes that could be detected in rivers.

**Table S7 Transport capacity, environmental filtration, and transportation effectiveness of watershed biological information flow (WBIF) from the riparian sampling site to adjacent riverine water sampling site in each sampling transect on summer rainy days, estimated at the species level**

|  | **Bacteria (detected by the 16S rRNA gene)** | | | **Fungi (detected by the ITS gene)** | | | **Eukaryotes (detected by the CO1 gene)** | | |
| --- | --- | --- | --- | --- | --- | --- | --- | --- | --- |
|  | **Transport capacity** | **Environmental filtration** | **Transportation effectiveness** | **Transport capacity** | **Environmental filtration** | **Transportation effectiveness** | **Transport capacity** | **Environmental filtration** | **Transportation effectiveness** |
| **S-W_SL6b** | 0.683468 | 0.021696 | 0.668639 | 0.625000 | 0.111111 | 0.555556 | 0.437819 | 0.245501 | 0.330334 |
| **S-W_SL6** | 0.874088 | 0.012613 | 0.863063 | 0.827907 | 0.092827 | 0.751055 | 0.600000 | 0.286492 | 0.428105 |
| **S-W_SL5** | 0.844639 | 0.033827 | 0.816068 | 0.591503 | 0.133144 | 0.512748 | 0.504570 | 0.313676 | 0.346299 |
| **S-W_SL5m** | 0.893401 | 0.026680 | 0.869565 | 0.850467 | 0.085470 | 0.777778 | 0.664207 | 0.306905 | 0.460358 |
| **S-W_SL4** | 0.880989 | 0.029257 | 0.855214 | 0.833948 | 0.117264 | 0.736156 | 0.700000 | 0.245283 | 0.528302 |
| **S-W_SL4b** | 0.814159 | 0.032120 | 0.788009 | 0.774359 | 0.129464 | 0.674107 | 0.619165 | 0.257299 | 0.459854 |
| **S-W_SL3** | 0.818637 | 0.032946 | 0.791667 | 0.669643 | 0.125000 | 0.585938 | 0.704082 | 0.313084 | 0.483645 |
| **S-W_SL2** | 0.760110 | 0.074043 | 0.703830 | 0.828244 | 0.176101 | 0.682390 | 0.627685 | 0.433784 | 0.355405 |
| **S-W_SL1** | 0.442045 | 0.081420 | 0.406054 | 0.594118 | 0.162562 | 0.497537 | 0.271255 | 0.433486 | 0.153670 |

S-W denotes the WBIF from the riparian soil eDNA sample (riparian zone) to the riverine water eDNA sample (river). SL1, SL2, SL3, SL4, SL4b, SL5m, SL5, SL6, and SL6b denote the sampling transects. S-W_SL1 denotes the WBIF from the riparian zone to the river at SL1. Bacteria (detected by the 16S rRNA gene), fungi (detected by the ITS gene), and eukaryotes (detected by the CO1 gene) indicate the groups of bacteria (detected by the 16S rRNA gene), fungi (detected by the ITS gene), and eukaryotes (detected by the CO1 gene), respectively. Transport capacity indicates the proportion of riparian soil microbes that were transported into the river. Environmental filtration indicates the proportion of riparian soil microbes that could not be kept alive in rivers. Transportation effectiveness indicates the proportion of riparian soil microbes that could be detected in rivers.

**Table S8 The accumulative runoff distance and accumulative transportation effectiveness from the first sampling transect in each chain of the upstream-to-downstream watershed biological information flow (WBIF) on summer rainy days, estimated at the OTU level**

| **Transport Chain** | **Cumulative**  **Distance/km** | **Cumulative Effectiveness** | | |
| --- | --- | --- | --- | --- |
|  |  | **Bacteria (detected by the 16S rRNA gene)** | **Fungi (detected by the ITS gene)** | **Eukaryotes (detected by the CO1 gene)** |
| **Chain A** |  |  |  |  |
| **SL6b-SL5** | 8 | 0.798283 | 0.549296 | 0.414278 |
| **##-SL4** | 23.5 | 0.534830 | 0.386642 | 0.274202 |
| **##-SL3** | 47 | 0.486959 | 0.330759 | 0.199567 |
| **##-SL2** | 62 | 0.383295 | 0.299409 | 0.173607 |
| **##-SL1** | 69 | 0.197425 | 0.220354 | 0.064900 |
| **Chain B** |  |  |  |  |
| **SL6-SL5** | 9 | 0.762412 | 0.576717 | 0.437427 |
| **##-SL4** | 24.5 | 0.590463 | 0.447964 | 0.248343 |
| **##-SL3** | 48 | 0.517251 | 0.365282 | 0.197661 |
| **##-SL2** | 63 | 0.414025 | 0.329905 | 0.161404 |
| **##-SL1** | 70 | 0.192146 | 0.223365 | 0.054191 |
| **Chain C** |  |  |  |  |
| **SL5m-SL4** | 8.5 | 0.765880 | 0.651294 | 0.625714 |
| **##-SL3** | 32 | 0.579820 | 0.445819 | 0.366984 |
| **##-SL2** | 47 | 0.448004 | 0.388826 | 0.265397 |
| **##-SL1** | 54 | 0.196740 | 0.237720 | 0.058413 |
| **Chain D** |  |  |  |  |
| **SL4b-SL3** | 23 | 0.738029 | 0.569938 | 0.518391 |
| **##-SL2** | 38 | 0.555864 | 0.466098 | 0.329119 |
| **##-SL1** | 45 | 0.237335 | 0.272641 | 0.068966 |

Chains A, B, C, and D denote the 4 chains of the upstream-to-downstream WBIF. In Chain A, SL6b-SL5 denotes the WBIF from sampling transects SL6b to SL5; ##-SL4 denotes the WBIF from sampling transects SL6b to SL5, and then to SL4; ##-SL3 denotes the WBIF from sampling transects SL6b to SL5, SL4, and then to SL3; ##-SL2 denotes the WBIF from sampling transects SL6b to SL5, SL4, SL3, and then to SL2; ##-SL1 denotes the WBIF from sampling transects SL6b to SL5, SL4, SL3, SL2, and then to SL1 (estuary). Bacteria (detected by the 16S rRNA gene), fungi (detected by the ITS gene), and eukaryotes (detected by the CO1 gene) indicate the groups of bacteria (detected by the 16S rRNA gene), fungi (detected by the ITS gene), and eukaryotes (detected by the CO1 gene), respectively.

**Table S9 The accumulative runoff distance and accumulative transportation effectiveness from the first sampling transect in each chain of the upstream-to-downstream watershed biological information flow (WBIF) on summer rainy days, estimated at the species level**

| **Transport Chain** | **Cumulative**  **Distance/km** | **Cumulative Effectiveness** | | |
| --- | --- | --- | --- | --- |
|  |  | **Bacteria (detected by the 16S rRNA gene)** | **Fungi (detected by the ITS gene)** | **Eukaryotes (detected by the CO1 gene)** |
| **Chain A** |  |  |  |  |
| **SL6b-SL5** | 8 | 0.897101 | 0.757895 | 0.515347 |
| **##-SL4** | 23.5 | 0.759420 | 0.701754 | 0.384491 |
| **##-SL3** | 47 | 0.711594 | 0.677193 | 0.312601 |
| **##-SL2** | 62 | 0.610870 | 0.659649 | 0.280291 |
| **##-SL1** | 69 | 0.377536 | 0.557895 | 0.137318 |
| **Chain B** |  |  |  |  |
| **SL6-SL5** | 9 | 0.862913 | 0.634091 | 0.501928 |
| **##-SL4** | 24.5 | 0.775398 | 0.590909 | 0.322865 |
| **##-SL3** | 48 | 0.712974 | 0.534091 | 0.271074 |
| **##-SL2** | 63 | 0.616891 | 0.522727 | 0.229201 |
| **##-SL1** | 70 | 0.355569 | 0.422727 | 0.099725 |
| **Chain C** |  |  |  |  |
| **SL5m-SL4** | 8.5 | 0.868212 | 0.787819 | 0.670061 |
| **##-SL3** | 32 | 0.753508 | 0.595285 | 0.454684 |
| **##-SL2** | 47 | 0.640635 | 0.563851 | 0.341650 |
| **##-SL1** | 54 | 0.356925 | 0.408644 | 0.112016 |
| **Chain D** |  |  |  |  |
| **SL4b-SL3** | 23 | 0.848338 | 0.688679 | 0.601319 |
| **##-SL2** | 38 | 0.715374 | 0.646226 | 0.414868 |
| **##-SL1** | 45 | 0.393352 | 0.457547 | 0.126499 |

Chains A, B, C, and D denote the 4 chains of the upstream-to-downstream WBIF. In Chain A, SL6b-SL5 denotes the WBIF from sampling transects SL6b to SL5; ##-SL4 denotes the WBIF from sampling transects SL6b to SL5, and then to SL4; ##-SL3 denotes the WBIF from sampling transects SL6b to SL5, SL4, and then to SL3; ##-SL2 denotes the WBIF from sampling transects SL6b to SL5, SL4, SL3, and then to SL2; ##-SL1 denotes the WBIF from sampling transects SL6b to SL5, SL4, SL3, SL2, and then to SL1 (estuary). Bacteria (detected by the 16S rRNA gene), fungi (detected by the ITS gene), and eukaryotes (detected by the CO1 gene) indicate the groups of bacteria (detected by the 16S rRNA gene), fungi (detected by the ITS gene), and eukaryotes (detected by the CO1 gene), respectively.
